# Supplementary material for: Nanoconfinement steers nonradical pathway transition in single atom fenton-like catalysis for improving oxidant utilization
Source: Nat Commun. 2024 Jun 22;15:5314. doi: 10.1038/s41467-024-49605-2 (PMC11192908; doi:10.1038/s41467-024-49605-2)
Supplement: Supplementary file 1 — Supplementary Information [file 41467_2024_49605_MOESM1_ESM.pdf]

Supplementary Information

Nanoconfinement Steers Nonradical Pathway Transition in Single Atom Fenton-like Catalysis for Improving Oxidant Utilization

Yan Meng,[1, 3†] Yu-Qin Liu,[1†] Chao Wang,[2†] Yang Si,[4] Yun-Jie Wang,[1, 3] Wen-Qi Xia,[1, 3] Tian Liu,[3] Xu Cao,[1] Zhi-Yan Guo,[1, 3*] Jie-Jie Chen,[1] Wen-Wei Li[1, 3*]

[1]CAS Key Laboratory of Urban Pollutant Conversion, Department of Environmental Science and Engineering,

[2]National Synchrotron Radiation Laboratory,

University of Science & Technology of China, Hefei 230026, China

[3]Sustainable Energy and Environmental Materials Innovation Center, Suzhou Institute for Advanced Research,

University of Science & Technology of China, Suzhou 215123, China

[4]Kunming Institute of Physics, Kunming 650223, China

[†]These authors contributed equally: Yan Meng, Yu-Qin Liu, Chao Wang.

[*]Corresponding Authors:

Dr. Zhi-Yan Guo, E-mail: gzy2018@ustc.edu.cn

Prof. Wen-Wei Li, E-mail: wwli@ustc.edu.cn

1. Supplementary Note

1.1 Analytical methods of catalytic activity

The pollutant concentration was measured by Agilent HPLC coupled with C_{18} (250 mm × 4.6 mm, 5μm) and UV detector. The methanol-water mixture was used as the mobile phase and operated at a constant flow rate of 1 mL/min. The volume ratio of MeOH/water and absorption wavelength (λ) information is listed in Supplementary Table 9. For the electron paramagnetic resonance (EPR) analysis, 5,5-dimethyl-1-pyrroline-N-oxide (DMPO) and 2,2,6,6-tetramethylpiperidine (TEMP) were employed as the spin-trapping agent to capture $SO_4^{\bullet-}$/•OH, $O_2^{-}\cdot$ and $^1O_2$, respectively. The corresponding signals were detected using a Bruker ER200-SRC EPR spectrometer. The PMS concentrations were determined by colorimetric method, based on a spectral detection of iodine (at 410 nm) as the major product of PMS-iodide reaction[1]. The $O_2\cdot^-$ was analyzed by using nitrotetrazolium blue chloride (NBT) as a probe for spectral detection with a UV-1800 spectrometer. The $^1O_2$ concentration was measured by using 1,3-diphenylisobenzofuran (DPBF) probe at 410 nm wavelength with a UV-1800 spectrometer. Raman spectra of CoNC-MSi -PMS* were measured with a green laser at 532 nm (LabRAM Horiba). The metal leaching amount was quantified by microwave plasma atomic emission spectroscopy (MP–AES4107, Agilent). The total organic carbon (TOC) was measured on TOC analyser (TOC-II, elementar).

1.2 Evaluation of catalytic activity

The initial pH values (before PMS addition) of the reaction solutions were adjusted using 0.1 M NaOH or HCl. In the cyclic degradation experiments, the CoNC-MSi catalysts were washed with DI water and ethanol after each cycle, then resuspended in the reaction solution for the next cycle operation. All the experiments were carried out in duplicate or triplicate.

The 4-CP degradation rates were calculated according to the pseudo-first-order kinetic model (Eq. (S1)).

$$-\ln\frac{C_{\mathrm{t}}}{C_0} = k_{\mathrm{obs}}\cdot t \qquad \text{(S1)}$$

where C_t is the 4-CP concentration at a certain reaction time (t), and C_0 is the initial 4-CP concentration (after reaching adsorption-desorption equilibrium); k_{obs} is the apparent degradation rate constant.

Normalizing the k_{obs} to the specific surface area ($S_{BET\ surface}$) of the catalysts yields the specific activity k_{SA} (g m^{-2} min^{-1}), which represents the intrinsic activity of a catalyst (Eq. (S2)).

$$k_{SA} = \frac{k_{obs}}{S_{BET\ surface}} \tag{S2}$$

The turnover frequency (TOF) of the Co SAC was calculated by normalizing the degradation rate constant to the single-atom Co content (the predominant reactive site) (Eq. (S3))

$$TOF = \frac{N_{4\text{-CP}}}{M_{Co\ SA}} \cdot t \tag{S3}$$

where $N_{4\text{-CP}}$ is the amount of removed 4-CP (mol) during a certain period, $M_{Co\ SA}$ is the mass of single-atom Co in the catalysts (g), and t is the total reaction time (h).

1.3 Identification and quantification of reactive species

In the radical quenching experiments, ethanol (EtOH) was used as the scavenger for •OH and $SO_4^{•-}$, tertiary butyl alcohol (TBA) for •OH, methylene blue (MB) for surface-bound radicals, and furfuryl alcohol (FFA) for $^1O_2$[2]. In addition, we replaced the water solution with D_2O to further validate the $^1O_2$ production. The contributions of •OH, $SO_4^{•-}$, $^1O_2$, surface-bound •OH and $SO_4^{•-}$, and Co^{IV}, for the 4-CP degradation were quantified based on the discrepancy of degradation kinetic constant in the presence of different scavengers. The apparent rate constants with EtOH, FFA, MB, DMSO addition, or at initial acid condition were denoted as k_1, k_2, k_3, and k_4, respectively, and the rate constant of the group without scavengers was k_0. Thus, the fractions of 4-CP degradation contributed by the above species and by direct oxidation at the catalyst surface were calculated according to (Eqs. (4)- (S7))[3].

$$\lambda(\bullet OH \text{ and } SO_4^{\bullet -}) = \frac{k_0\text{-}k_1}{k_0} \tag{S4}$$

$$\lambda(^1O_2) = \frac{k_0\text{-}k_2}{k_0} \tag{S5}$$

$$\lambda(\text{surface-bound } \bullet OH \text{ and } SO_4^{\bullet -}) = \frac{k_0\text{-}k_3}{k_0} \tag{S6}$$

$$\lambda(Co^{IV}) = \frac{k_0\text{-}k_4}{k_0} \tag{S7}$$

1.4 Calculation of PMS utilization efficiency for 4-CP degradation

The PMS utilization efficiency (PUE) was estimated following the rules reported in a previous study[4]. Here, the *PUE* for 4-CP degradation is defined as the ratio of the PMS used for 4-CP mineralization ($n_{\text{PMS mineralization}}$, mol) to the decomposed PMS amount ($n_{\text{PMS decomposition}}$, mol), as shown in (Eq. (S8)):

$$PUE = \frac{n_{\text{PMS, mineralization}}}{n_{\text{PMS, decomposition}}} \tag{S8}$$

The half-reactions for 4-CP mineralization and PMS activation are described in Eqs. (S9) and Eqs. (S10) respectively. In theory, a complete mineralization of one 4-CP molecule would loss 26 electrons, while one mole PMS molecule ($H_3K_5O_{18}S_4$) can accept 14 electrons to generate anions. The overall reactions for 4-CP mineralization by PMS (ignoring the activation process) is shown in (Eqs. (S11))[5].

$$C_6H_5OCl + 11H_2O - 26e^- \rightarrow 6CO_2 + Cl^- + 27H^+ \tag{S9}$$

$$H_3O_{18}S_4^{5-} + H^+ + 14e^- \rightarrow 2H_2O + 4SO_4^{2-} \tag{S10}$$

$$14C_6H_5OCl + 26H_3K_5O_{18}S_4 \rightarrow 84CO_2 + 28H_2O + 14HCl + 65K_2SO_4 + 39H_2SO_4 \tag{S11}$$

Similarly, a complete mineralization of one mole phenol (PhOH), bisphenol A (BPA), p-nitrophenol (4-NP), chlorobenzene (CB) and benzoic acid (BA) will theoretically consume 7, 18, 8.5, 7 and 7.5 moles of PMS, as expressed in (Eqs. (S12)- (S16)).

$$2C_6H_6O + 14H_3K_5O_{18}S_4 \rightarrow 12CO_2 + 6H_2O + 35K_2SO_4 + 21H_2SO_4 \tag{S12}$$

$$C_{15}H_{16}O_2 + 18H_3K_5O_{18}S_4 \rightarrow 15CO_2 + 8H_2O + 45K_2SO_4 + 27H_2SO_4 \tag{S13}$$

$$4C_6H_5NO_2 + 34H_3K_5O_{18}S_4 \rightarrow 24CO_2 + 8H_2O + 4HNO_3 + 51K_2SO_4 + 85H_2SO_4 \tag{S14}$$

$$2C_6H_5Cl + 14H_3K_5O_{18}S_4 \rightarrow 12CO_2 + 4H_2O + 2HCl + 21K_2SO_4 + 35H_2SO_4 \tag{S15}$$

$$4C_7H_6O_2 + 30H_3K_5O_{18}S_4 \rightarrow 28CO_2 + 12H_2O + 45K_2SO_4 + 75H_2SO_4 \tag{S16}$$

According to Eq. (S8), $n_{PMS,\ mineralization}$ can be calculated from the TOC changes (corresponding to pollutant mineralization) (mol, Eqs. (S11)- (S16)). The $n_{PMS,\ decomposition}$ (i.e., the amount of decomposed PMS) can be estimated by traditional UV colorimetric method at 410 nm using UV-1100 spectrophotometer.

1.5 Galvanic cell assay for identifying electron transfer pathway

A dual-chamber galvanic cell was constructed for the test. The electrode was prepared by dripping 1 mL of the catalyst suspension (pretreated by ultrasonic dispersion of 40 mg catalysts in 2 mL isopropanol) on both external surfaces of a carbon flake (4.5 cm×4.5 cm) and dried at 50℃ in an oven. The two electrolyte chambers were separated by the electrode and externally connected through an agar salt bridge. The latter was prepared by feeding a preheated mixture of 1.5 M $NaNO_3$, 0.3 g of agar (1.5%) and 30 mL water into a U-shaped tube, followed by naturally cooling down to room temperature. The two electrolyte chambers were added with 0.05 mM 4-CP and 0.8 mM PMS respectively to initiate the galvanic cell reactions.

1.6 Electrochemical assays

The electrochemical assays were performed using a CHI760E electrode workstation (Chenhua Instrument Co., China) with a three-electrode system. The catalyst suspension was prepared by adding 5 mg catalysts into a solution containing 2 mL isopropanol and 20 μL Nafion under ultrasonication. Then, the catalyst suspension was loaded onto a clean glassy carbon electrode through dip coating and dried at room temperature, and this procedure was repeated twice. The resulting electrode was used as the working electrode. The platinum wire and Ag/AgCl (filled with saturated KCl) was used as the counter and reference electrode, respectively. The electrodes were inserted in 40 mL Na_2SO_4 electrolyte (10 mM) in a 100 mL breaker. The open circuit potential (OCP) was measured following a reported method[2]. In addition, the *i*-*t* curve was measured by chronoamperometry. Specifically, after applying the voltage for 400 s, 0.4 mM PMS was added, followed by adding 0.1 mM 4-CP. Then, electrochemical impedance spectroscopy (EIS) was performed using the electrolyte containing 10 mM KCl, 5 mM potassium ferricyanide and potassium ferrocyanide. The EIS measurement was conducted under the OCP condition with an amplitude of 10 mV, and initial and final frequencies were set as 5 MHz and 10 Hz, respectively.

1.7 Toxicity assessment

The cytotoxicity of 4-CP and its oxidation intermediates was evaluated by *E. coli* activity[6]. The solution containing 0.1 mM 4-CP before or after 30 min reaction was used as the feed solution. The aseptic water was used as the blank control. Each 0.9 mL solution was mixed with 0.1 mL *E. coli* suspension (10^9 CFU/mL), and then incubated at 37 ºC for 6 h. Then, the sample was transferred to a solid agar medium and incubated at 37 ℃ for 24 h prior to colony counting. The same toxicity evaluation method was used for measuring the treatment performance of the continuous-flow system.

1.8 Pollutant decontamination by CoNC catalytic membrane

A circular ceramic membrane loaded with CoNC was fabricated and its catalytic activity for 4-CP removal was investigated using a dead-end filtration device. The effective membrane area was 3.94 cm^2. The membrane module was operated at a constant water flux of 200 L m^{-2} h^{-1} driven by a peristaltic pump. Prior to the degradation experiment, the membrane was subjected to pre-adsorption with 4-CP for ~2 hours to achieve the adsorption equilibrium. Then, PMS was added in the feedwater to initiate the degradation reaction and the transmembrane pressure difference was recorded every 30 minutes by a pressure sensor.

2 Supplementary Table

Supplementary Table 1. BET surface area, average pore diameter, and pore volume of the prepared samples

Catalyst	BET surface area (m^2 g^{-1})	Pore diameter (nm)	Pore volume (cm^3 g^{-1})
CoNC-MSi1	85.89	7.71	0.15
CoNC-MSi2	74.27	12.83	0.21
CoNC-Si	14.48	/	/

Supplementary Table 2. The Co contents relative to the shell mass or to the total mass of different catalysts

Catalyst		CoNC-MSi1	CoNC-MSi2	CoNC-Si
Co content[a] (wt%)	Relative to the shell mass	3.8 ± 0.3	1.2	0.2 ± 0.1
	Relative to the total mass	0.07	0.05 ± 0.01	0.01

a. All the Co loading amount was assessed twice by ICP-AES measurement.

The core-shell structured porous silica particle with high mass density was used as the support but Co single atoms were confined on the porous shell, resulting in a low value of SAC loading relative to the total mass of the catalyst.

Supplementary Table 3. Proportions of different N species in the catalysts determined by N 1*s* XPS spectra

Catalyst	Pyridinic N	Co-N	Pyrrolic N	Graphitic N	Oxidized N
CoNC-MSi1	0.14	0.24	0.31	0.31	0
CoNC-MSi2	0.12	0.24	0.23	0.41	0
CoNC-Si	0.16	0.17	0.17	0.45	0.05

Supplementary Table 4. Fractions of the key elements in the catalyst based on XPS spectra results

Catalyst	Spectrum peak	Atomic ratio [%]	Mass ratio [%]
CoNC-MSi1	Co 2*p*	0.2	0.7
	N 1*s*	1.7	1.6
	Si 2*p*	11.7	21.6
	O 1*s*	28.6	30.2
	C 1*s*	57.9	45.9
CoNC-MSi2	Co 2*p*	0.1	0.5
	N 1*s*	1.2	1.0
	Si 2*p*	17.0	29.1
	O 1*s*	39.6	38.6
	C 1*s*	42.0	30.7
CoNC-Si	Co 2*p*	0.1	0.3
	N 1*s*	0.6	0.5
	Si 2*p*	21.7	34.5
	O 1*s*	52.4	47.6
	C 1*s*	25.2	17.2

Supplementary Table 5. Structural parameters of the catalysts extracted from the Co *K*-edge EXAFS fitting

Catalyst	shell	***CN*[a]**	***R* (Å)[b]**	**σ^2 (10^3Å^2)[c]**	**ΔE_0 (eV)[d]**	***R* factor**
CoPc	Co-N	4	1.90 (±0.02)	2.0 (±3.3)	2.2 (±5.0)	0.034
CoNC-MSi1	Co-N	4.5 (±0.5)	1.93 (±0.02)	3.0*	-14.5 (±3.2)	0.013
CoNC-MSi2	Co-N	4.9 (±1.3)	1.78 (±0.04)	3.0*	-38.5 (±14.3)	0.100
CoNC-Si	Co-N	3.1 (±0.8)	1.89 (±0.03)	3.0*	-10.0 (±9.5)	0.058

CN[a], coordination number; *R*[b], the distance between absorber and backscatter atoms; σ^{2}[c], Debye-Waller factor to account for both thermal and structural disorders; ΔE_0[d], inner potential correction; *R* factor indicates the goodness of the fit.

Supplementary Table 6. Comparison of decontamination performances with the state-of-the-art catalysts for nonradical-dominated Fenton-like catalysis

Catalyst (g L^{-1})	Pollutant	PMS	Reaction Pathway	BET surface area (m^2 g^{-1})	*k* (min^{-1})	$k_{per\text{-}site}$ (min^{-1} g^{-1})	k_{SA} (g m^{-2} min^{-1})	*PUE*[a] (%)	Ref.
NBC800 (0.5)	4-CP 100 mg L^{-1}	3.25 mM	*HSO_5^- nonradical	246	0.0375	/	0.00015	56.6	7
BSN-800 (0.1)	Gatifloxacin (GAT) 8 mg L^{-1}	0.4 g L^{-1}	$^1O_2$, ETP	419.1	0.123	/	0.0003	6.5	8
SWBC800 (0.1)	Tetracycline (TC) 10 mg L^{-1}	0.5 mM	$^1O_2$, $SO_4^{\cdot -}$	648.62	2.5	/	0.0039	36.5	9
Co/La-$SrTiO_3$ (1.0)	O-nitrophenol (ONP) 20 mg L^{-1}	0.4 g L^{-1}	$^1O_2$, $SO_4^{\cdot -}$	110.1	0.344	/	0.003	22.1	10
C-$CoMnO_x$ (0.1)	Bisphenol A (BPA) 10 mg L^{-1}	0.05 g L^{-1}	ETP	63.04	0.61	/	0.0097	39.4	11
Cu-In_2O_3/O_v (0.5)	TC 20 mg L^{-1}	1.0 mM	ETP, $SO_4^{\cdot -}$, ·OH	20.08	0.256	/	0.0125	9.5	12
Pd_4S (0.25)	4-CP 0.1 mM	0.25 mM	ETP	47.6	0.296	/	0.00625	78.6	2
Fe_1/CN (0. 5)	4-CP 0.1 mM	1.0 mM	$^1O_2$	80.66	0.55	9.82	0.0025	12	13
5-SAFe-CN (0.1)	Phenylphenol (OPP) 10 mg L^{-1}	0.4 g L^{-1}	ETP	28.03	0.1611	59.6	0.0057	31.4	14
Fe-SA/PHCNS (0.05)	acetaminophen (ACE) 10 mg L^{-1}	0.2 g L^{-1}	FeN_6=O	88.5	0.437	/	0.00495	50.8	15
ZMCs (0.05)	Acetaminophen (APAP) 0.1 mM	0.5mM	ETP	236.71	0.26	30.89	0.0011	25	16
Co-N_2 (0.2)	BPA 50 μM	2.0 mM	$^1O_2$, $SO_4^{\cdot -}$, ·OH	880.4	0.695	153.76	0.0008	6.3	17
CoN_1O_2 (0.2)	Sulfamethoxazole (SMX) 2 mg L^{-1}	0.2 mM	$Co^{(IV)}$=O	/	/	/	0.0008	9.9	18
CoN_3O_1 (0.1)	Ciprofloxacin (CIP) 5 mg L^{-1}	1 mM	$^1O_2$	67.64	0.287	93.18	0.0042	2.2	19
CoNC-Si (0.25)	4-CP 0.1 mM	0.4 mM	$^1O_2$	15.0	0.0545	2180	0.00375	61.8	This work
CoNC-MSi2 (0.25)	4-CP 0.1 mM	0.4 mM	$^1O_2$, ETP	41.7	1.32	10560	0.0315	82.7	This work
CoNC-MSi1 (0.25)	4-CP 0.1 mM	0.4 mM	ETP, $^1O_2$	50.5	1.95	11142.9	0.0385	96.6	This work

[a]. Due to missing of TOC or PMS decomposition data in the literature, the PUE values were approximated as the $\Delta n_{Pollutant}/\Delta n_{PMS}$ within a certain time period.

Supplementary Table 7. Adsorption energy (E_{ads}), bond length of adsorbed PMS (l_{O-O}, l_{S-O}, l_{O-H}, and l_{Co-O}), and corresponding charge transfer number (Q) from catalysts to PMS

Catalyst	E_{ads} (eV)		l_{O-O} (Å)	l_{S-O} (Å)	l_{O-H} (Å)	l_{Co-O} (Å)	Q
	PMS	4-CP					
CoNC-MSi1	-2.81	-2.42	1.47	1.67	0.98	2.10	0.84
CoNC-MSi2	-2.80	-2.38	1.47	1.67	0.98	2.10	0.82
CoNC-Si(CoN_3)	-2.56	-1.21	1.47	/	/	2.20	0.70
CoNC-Si(CoN_4)	-2.57	-1.20	1.47	1.66	0.98	2.20	0.77
CoNC-MSi3(2.7 nm)	-2.16	-2.00	1.48	1.70	0.99	/	0.43
Graphene	-0.14	/	1.48	1.70	0.99	/	0.94
Graphitic N	-0.45	/	1.48	1.71	0.99	/	0.64
Pyridinic/pyrrolic N	-0.26	/	1.48	1.71	0.99	/	0.93

Supplementary Table 8. PUE for pollutant degradation within 10 min in the CoNC-MSi1/PMS system

Pollutants	TOC removal (%)	$n_{PMS\ mineralization}$ (mM)	$n_{PMS\ decomposition}$ (mM)	PUE (%)
4-CP	51.6	0.097	0.1	96.6
PhOH	25.2	0.175	0.19	92.1
BPA	14.5	0.136	0.15	90.7
4-NP	9.4	0.020	0.03	65.3
BC	4.5	0.004	0.01	38.5
BA	4.5	0.002	0.01	21.0

Supplementary Table 9. Operating parameters for HPLC analysis of different analytes

Analyte	Flow (mL min^{-1})	λ (nm)	CH_3OH (%)	Acetonitrile (%)	H_2O (%)	Others (%)
4-CP	1	235	70	0	30	
PhOH	1	235	70	0	30	
BPA	1	250	70	0	30	
4-NP	1	262	0	65	0	35[a]
CB	1	225	70	0	30	
BA	1	230	10	0	0	90[b]
PMSO	0.8	230	0	70	30	
$PMSO_2$	0.8	215	0	70	30	

[a]. 3.85 g L^{-1} ammonium acetate solution + 3 g L^{-1} acetic acid solution
[b]. 0.2 M ammonium acetate solution.

3 Supplementary Figures

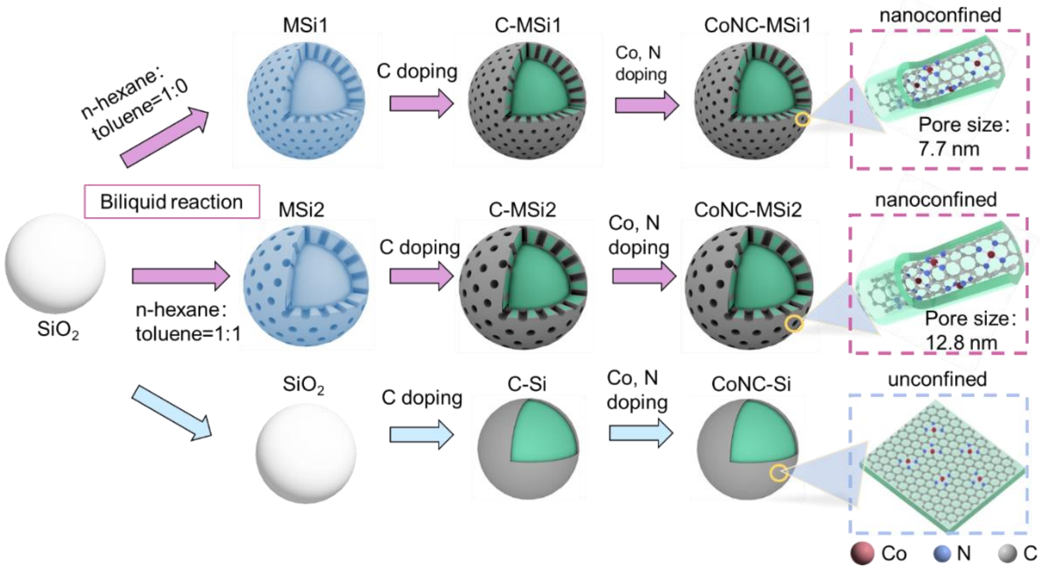

Supplementary Figure 1. Operating procedures for the catalyst synthesis. C precursor refers to the 2,3-Dihydroxynaphthalene, Co, N precursor refers to the Cobalt (II) acetate and 1,10-Phenanthroline, respectively.

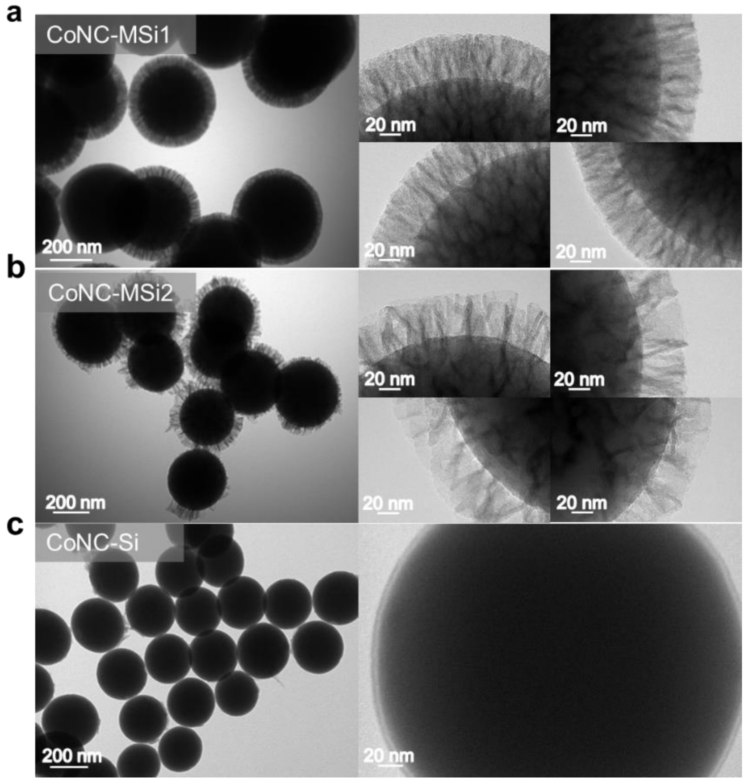

Supplementary Figure 2. Morphologies of the catalyst materials. TEM images of **(a)** CoNC-MSi1, **(b)** CoNC-MSi2 and **(c)** CoNC-Si at different magnification.

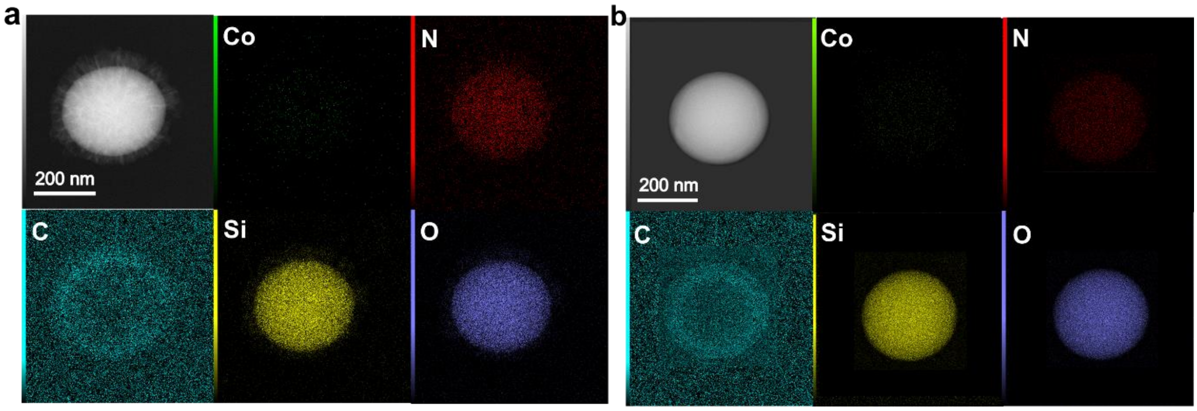

Supplementary Figure 3. Elemental distribution of the catalysts. TEM images and corresponding EDS mappings of **(a)** CoNC-MSi2 and **(b)** CoNC-Si.

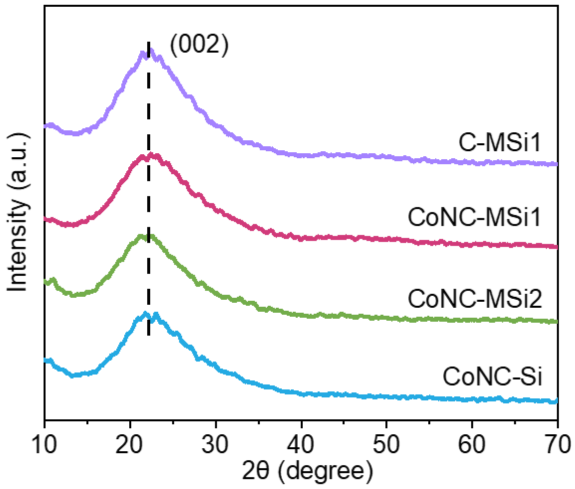

Supplementary Figure 4. X-ray diffraction (XRD) patterns of different catalysts.

The XRD patterns show no characteristic peaks of Co species in all catalysts, and only the peaks of the amorphous graphite carbon (002) were detected[20].

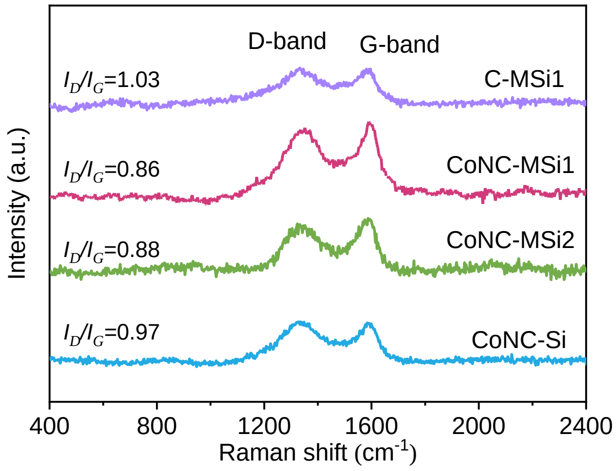

Supplementary Figure 5. Raman spectra of different catalysts.

No obvious Raman signals of Co-Co (463 cm^{-1}) or Co-O (1150 cm^{-1}) were detected in all the catalysts. Only two feature peaks of carbon, corresponding to the defect-induced D-band (~1300 cm^{-1}) and graphitic G-band (~1580 cm^{-1}) respectively, were detected. The ratio of D-band to G-band intensity (I_D/I_G) decreased by the order of CoNC-Si > CoNC-MSi2 > CoNC-MSi1, indicating a relatively higher graphitic degree and conductivity of CoNC-MSi1 (I_D/I_G = 0.86)[21].

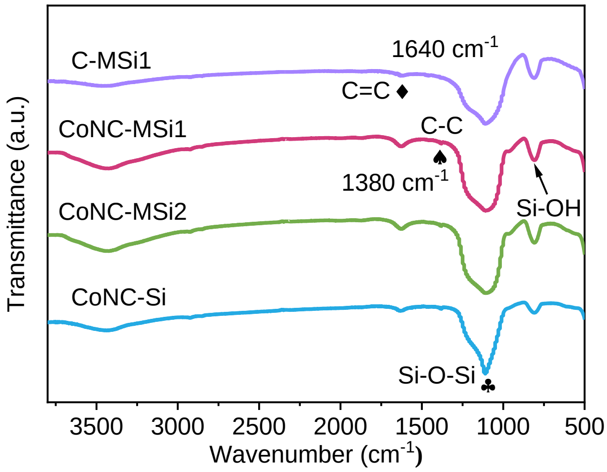

Supplementary Figure 6. FT-IR spectra of different catalysts.

No characteristic peaks of Co element were detected in all the catalysts. The peaks at 810, 1075, 1215, 1400-1700, and 3425 cm^{-1} were assigned to the bending vibrations of the Si-OH, the stretching vibration of Si-O-Si bond, the plane vibration of imidazole ring, the stretching vibration at imidazole ring and stretching vibration of O-H bond, respectively[22]. The results suggest the presence of carbon-wrapped MSi or SiO_2 and likely formation of Co single atoms.

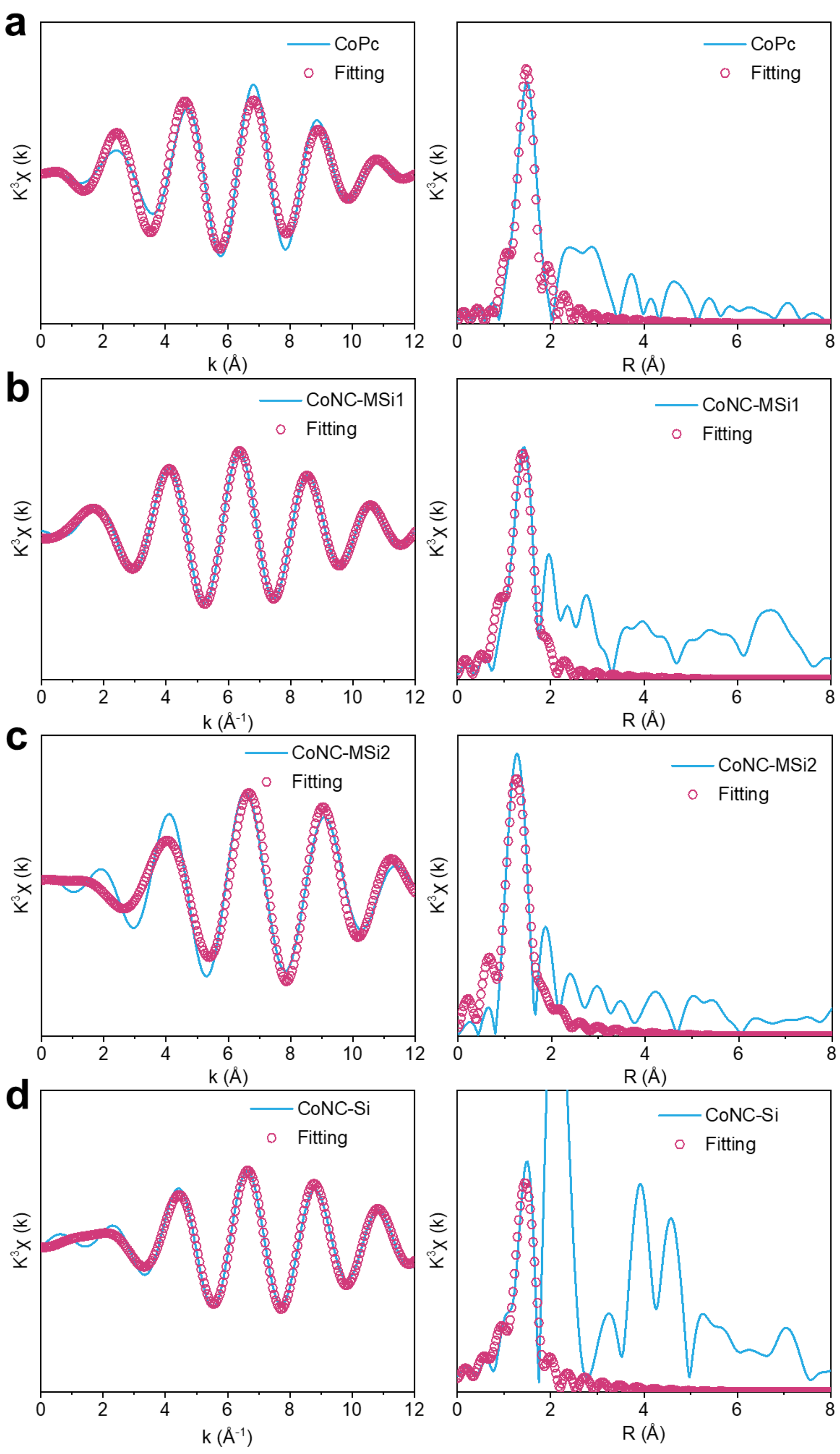

Supplementary Figure 7. Co *K*-edge EXAFS fitting plots. EXAFS fitting curves of **(a)** CoPc, **(b)** CoNC-MSi1, **(c)** CoNC-MSi2 and **(d)** CoNC-Si catalysts in *K*-space and *R*-space, respectively.

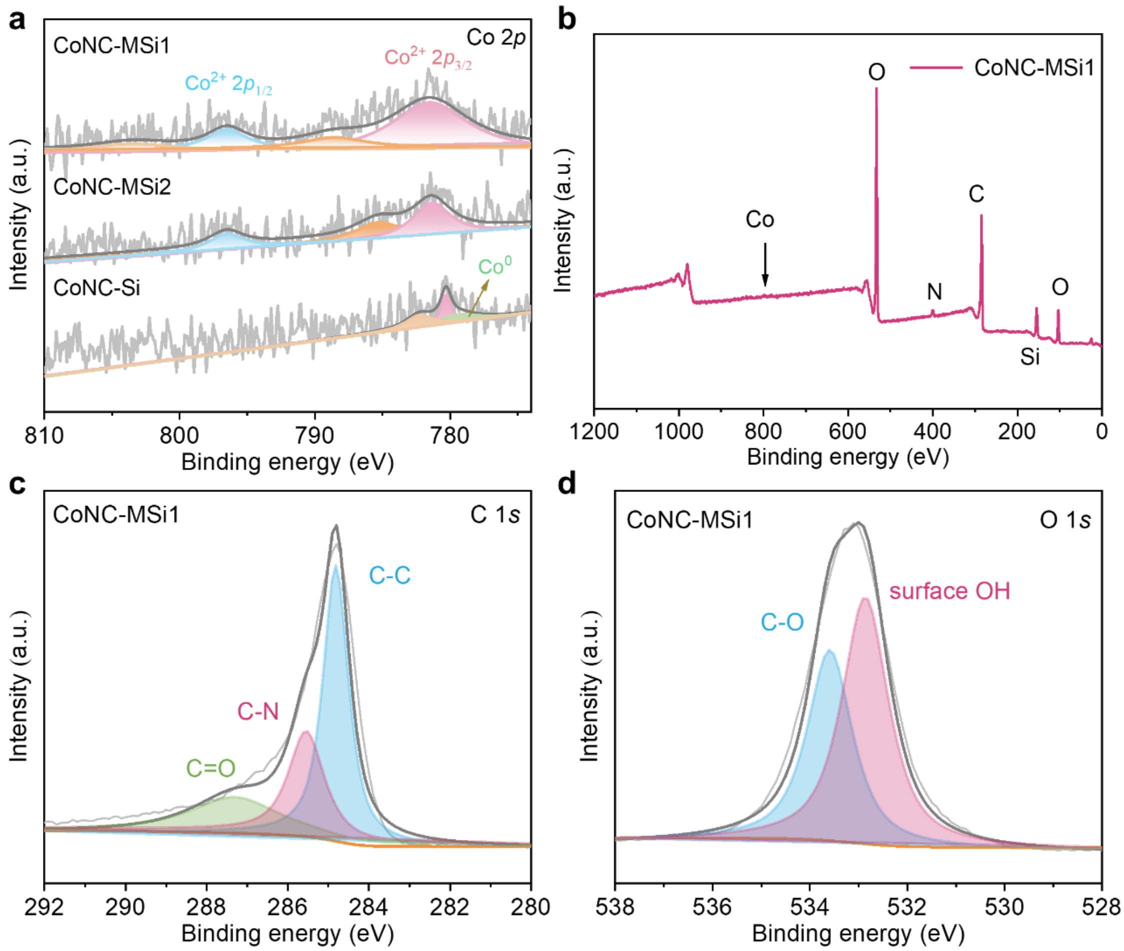

Supplementary Figure 8. XPS spectra of the catalysts. The XPS of **(a)** Co 2*p* spectrum for different catalysts, **(b)** the full survey scan, **(c)** C *1*s, and **(d)** O 1*s* of CoNC-MSi1.

Co $2p_{3/2}$ peak was observed in all the catalysts. The Co $2p_{3/2}$ and Co $2p_{1/2}$ peaks at binding energies of ~781 and 796 eV respectively suggest the presence of Co^{2+}. The Co^0 peak (778.8 eV) only appeared in CoNC-Si, with Co^0 content accounting for only ~3% of the total amount[23]. The C 1s and O1s spectra show distinct signals of C-C, C-N and C=O, indicating a successful formation of a carbon coating layer[24, 25].

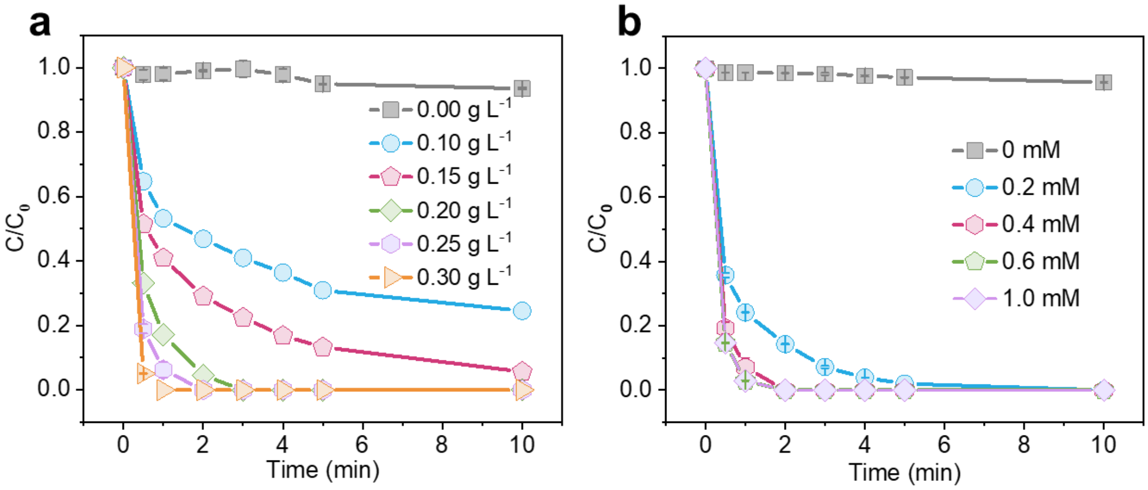

Supplementary Figure 9. 4-CP removal in the CoNC-MSi1/PMS system under different reactant dosages. **(a)** different catalyst dosage, **(b)** different PMS dosage. Error bars represent the standard deviation, obtained from two repeated experiments. Reaction conditions: [4-CP] =0.1 mM, [catalyst]= 0~0.30 g L^{-1}, [PMS]= 0-0.4 mM.

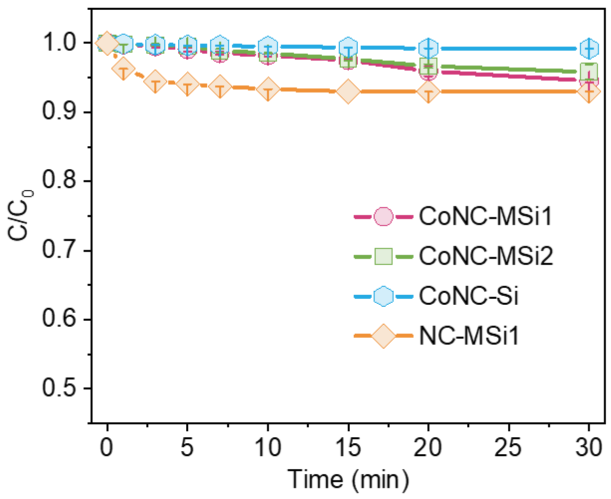

Supplementary Figure 10. 4-CP removal in the absence of PMS. Error bars represent the standard deviation, obtained from two repeated experiments. Reaction conditions: [catalysts] = 0.25 g L^{-1}, [4-CP] =0.1 mM.

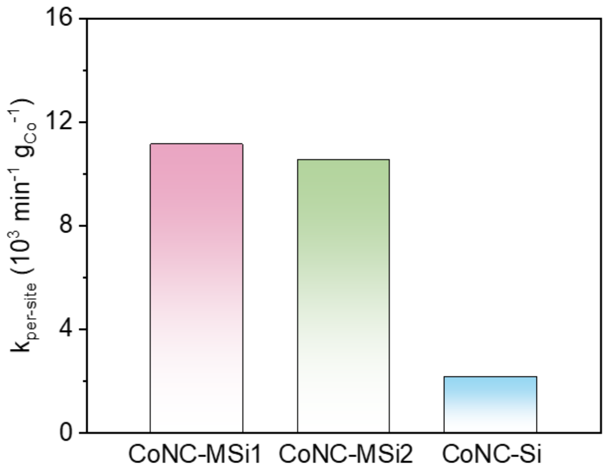

Supplementary Figure 11. Normalized kinetic constants ($k_{per\text{-}site}$) for 4-CP degradation to unit Co mass weight in different catalysts.

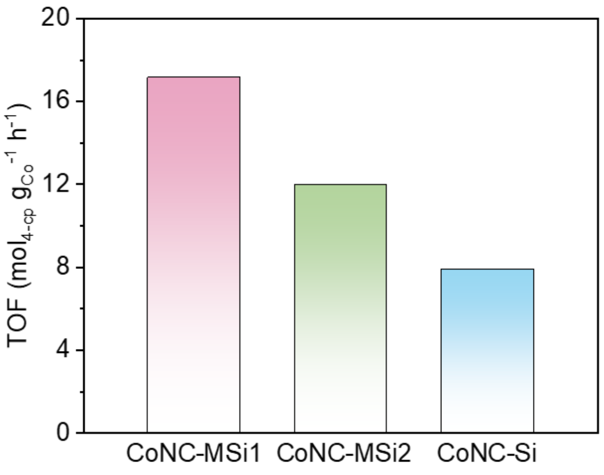

Supplementary Figure 12. Turnover frequency (TOF) for 4-CP degradation.

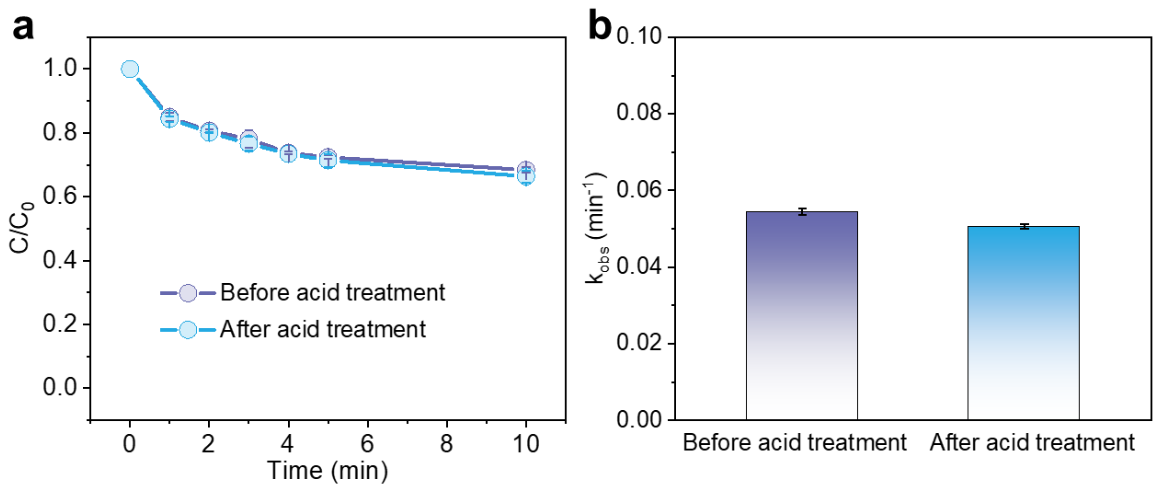

Supplementary Figure 13. Effect of acid treatment on 4-CP removal in CoNC-Si/PMS system. (a) 4-CP degradation profile and **(b)** corresponding kinetic constants before and after acid treatment. Error bars represent the standard deviation, obtained by repeating the experiment twice. Reaction conditions: [catalysts] = 0.25 g L^{-1}, [PMS] = 0.4 mM, [4-CP] =0.1 mM.

The reactivity of CoNC-Si remained almost unchanged after being washed with 0.1 M H_2SO_4 at 80°C for 6 hours, indicating a negligible role of Co atom clusters in the pollutant degradation.

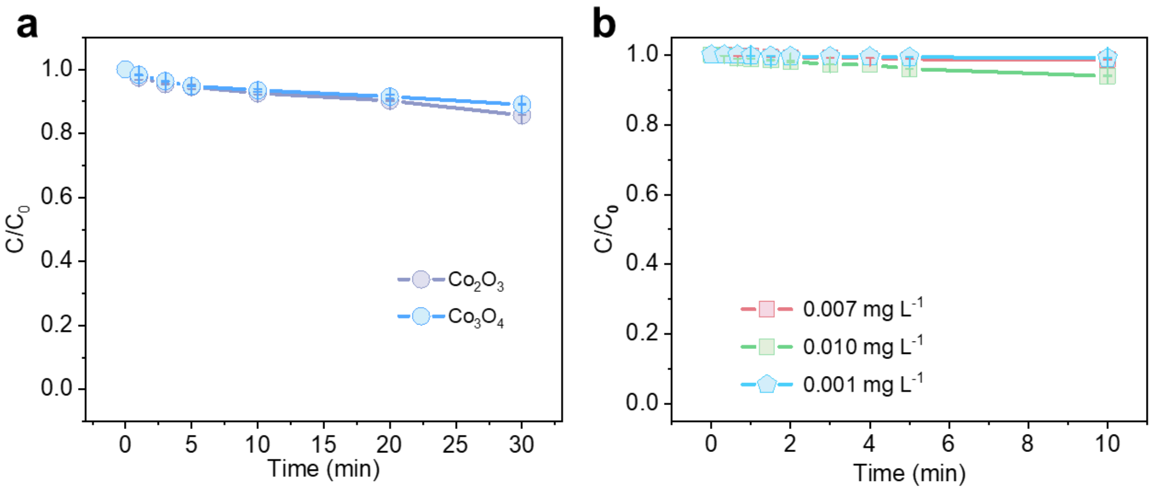

Supplementary Figure 14. 4-CP removal in Fenton-like catalysis systems with different cobalt oxides and Co^{2+} ions. (a) different cobalt oxide /PMS systems, **(b)** homogenous Co^{2+}/PMS systems with different Co^{2+} concentrations (equivalent to leached Co^{2+} amount from the heterogenous system). Error bars represent the standard deviation, obtained by repeating the experiment twice. Reaction conditions: [Co_3O_4] = [Co_2O_3] = 0.25 g L^{-1}, [PMS] = 0.4 mM, [4-CP] =0.1 mM.

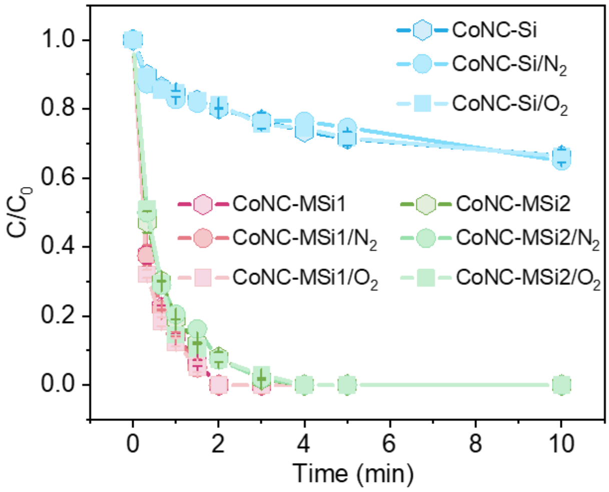

Supplementary Figure 15. 4-CP degradation performance under N_2 or O_2 purging conditions. Error bars represent the standard deviation, obtained by repeating the experiment twice. Reaction condition: [catalyst]= 0.25 $g \cdot L^{-1}$, [PMS] = 0.4 mM, [4-CP] = 0.1 mM, Gas purging time = 30 min.

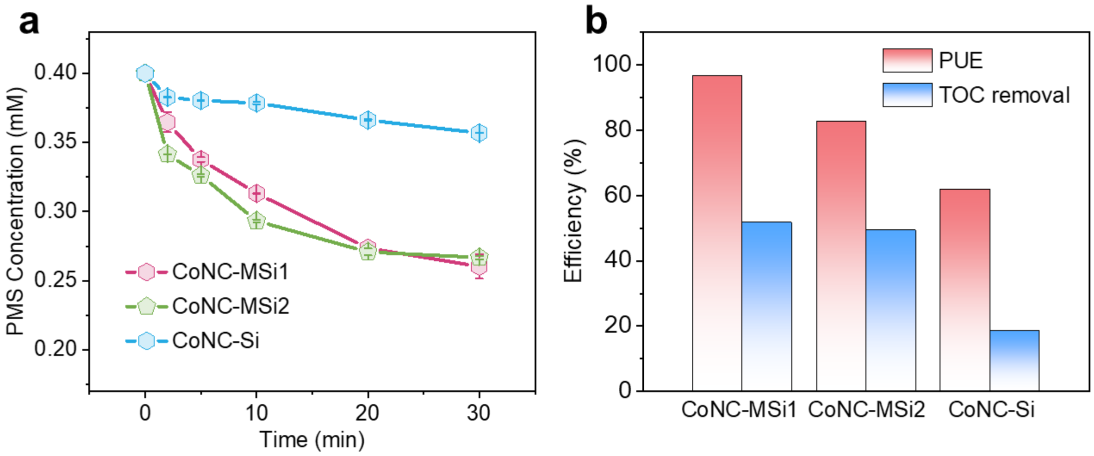

Supplementary Figure 16. PMS decomposition, PUE and TOC removal in different catalytic systems. (a) PMS concentration changes, **(b)** TOC removal and PUE within 10 minutes for 4-CP degradation. Error bars represent the standard deviation, obtained by repeating the experiment twice. Reaction conditions: [catalysts] = 0.25 g L^{-1}, [PMS] = 0.4 mM, [4-CP] = 0.1 mM.

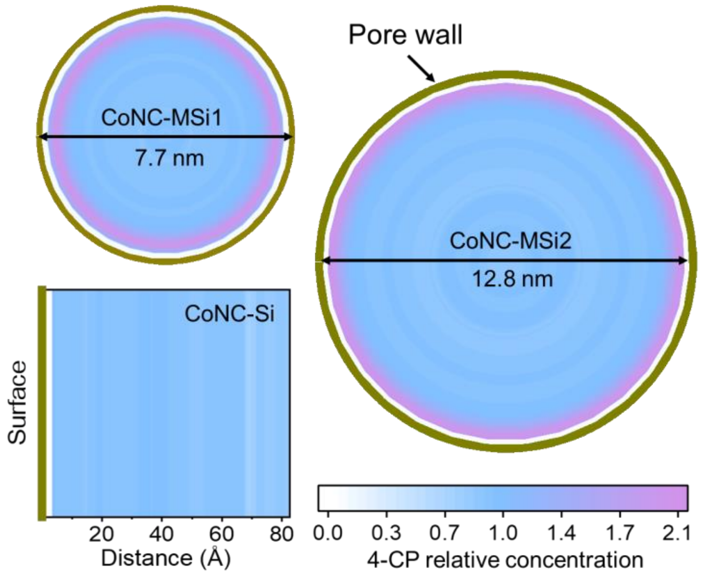

Supplementary Figure 17. Concentration distribution of 4-CP obtained by molecular dynamic simulations.

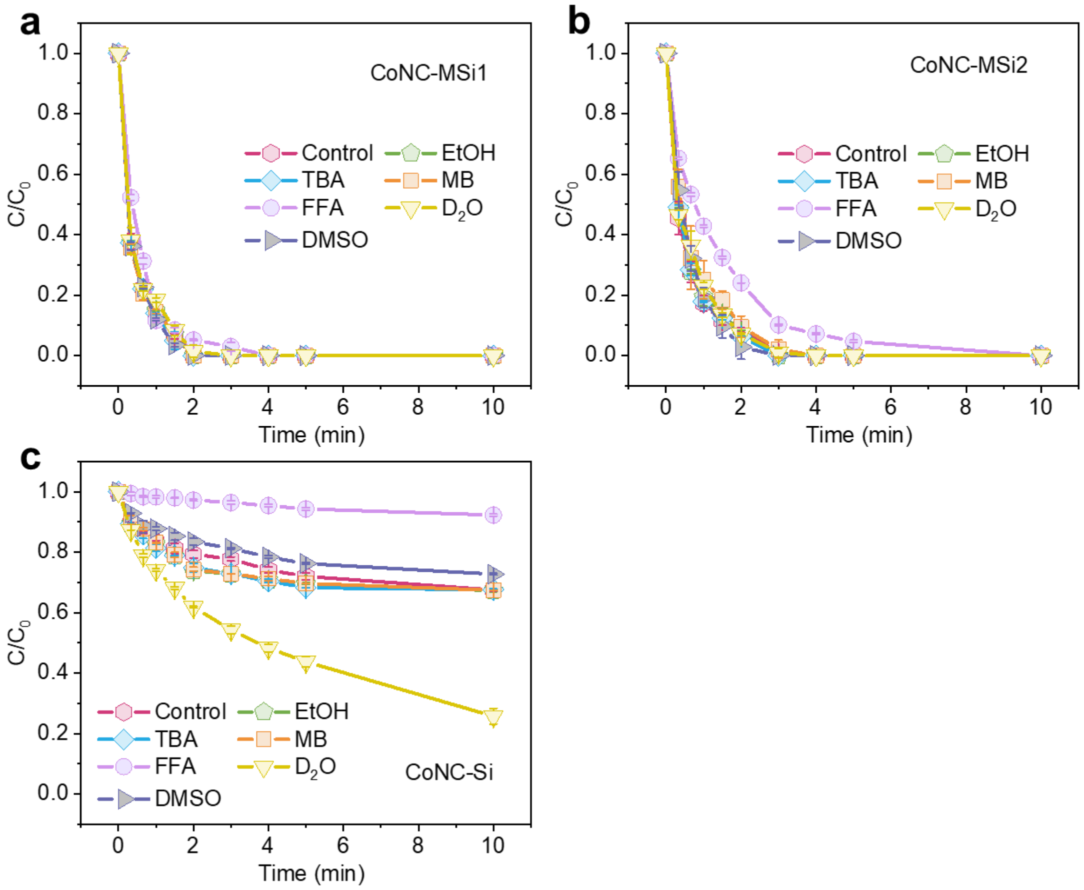

Supplementary Figure 18. 4-CP degradation in the presence of different scavengers. (a) CoNC-MSi1/PMS system, **(b)** CoNC-MSi2/PMS system, **(a)** CoNC-Si/PMS system. Error bars represent the standard deviation, obtained by repeating the experiment twice. Reaction conditions：[catalysts] = 0.25 g L^{-1}, [PMS] = 0.4 mM, [4-CP] =0.1 mM, [EtOH] = [TBA] = 400 mM, [MB] = 0.1 mM, [FFA] = 4 mM, [DMSO] = 0.5 mM.

The methanol (EtOH), tert-butanol (TBA), and methylene blue (MB) scavengers caused no obvious inhibition on 4-CP degradation in all the systems, implying no radicals ($SO_4^{\bullet-}$/•OH) were generated[2]. FFA (a $^1O_2$ quencher) slightly suppressed the 4-CP removal in the CoNC-MSi/PMS systems but caused severe inhibition in the CoNC-Si/PMS system. Replacing DI water with D_2O drastically accelerated the 4-CP degradation in the CoNC-Si/PMS system, confirming a predominant generation of $^1O_2$[26].

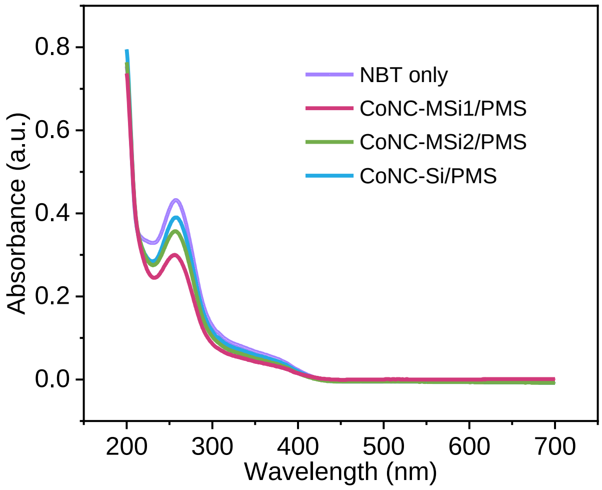

Supplementary Figure 19. UV-vis adsorption spectra of nitrotetrazolium blue chloride (NBT) in different systems. Reaction conditions: [catalysts] = 0.25 g L^{-1}, [PMS] = 0.4 mM.

No absorption peak of the product from reduction of NBT by $O_2^{\bullet-}$ (~520 nm) was observed[27], excluding the generation of $O_2^{\bullet-}$ in three catalysts/PMS systems.

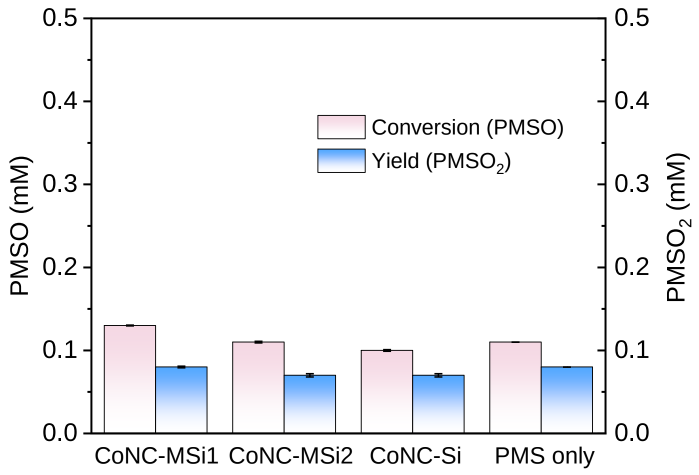

Supplementary Figure 20. PMSO conversion and concentration of generated $PMSO_2$ in different systems. Error bars represent the standard deviation, obtained by repeating the experiment twice. Reaction conditions: [catalysts] = 0.25 g L^{-1}, [PMS] = 0.4 mM, [PMSO] =0.5 mM.

The Co(II) or Co(II) complexes can react with PMS via two-electron transfer pathway to form high-valent Co(IV)-oxo complex for nonradical oxidation[28, 29]. Dimethyl sulfoxide (DMSO) and methyl phenyl sulfoxide (PMSO) were selected as the probes for Co(IV)-oxo species, and the corresponding sulfones would be selectively produced through Co(IV)-oxo species[30]. However, the 4-CP degradation was only slightly inhibited by DMSO addition (Supplementary Fig. 18) and the PMSO was not 100% oxidized to methyl phenyl sulfone ($PMSO_2$), indicating a negligible formation of Co(IV)-oxo species in the three catalysts/PMS systems.

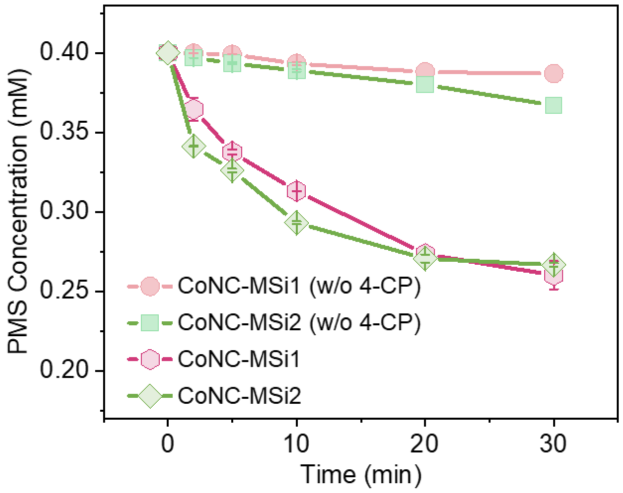

Supplementary Figure 21. PMS decomposition in nanoconfined catalytic systems. Error bars represent the standard deviation, obtained by repeating the experiment twice. Reaction conditions: [catalysts] = 0.25 g L^{-1}, [PMS] = 0.4 mM, [4-CP] =0.1 mM.

The PMS decomposition was drastically increased after 4-CP addition. Such pollutant-dependent PMS consumption behaviour was typical of catalytic system for decontamination via electron transfer process (ETP) pathway[31].

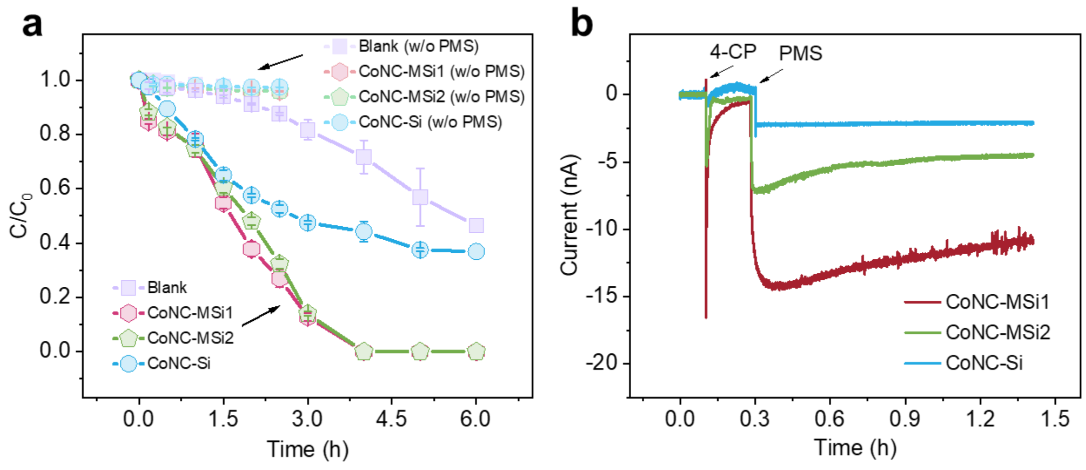

Supplementary Figure 22. 4-CP degradation performance in the dual-chamber galvanic cell. (a) 4-CP removal without and with PMS, **(b)** current changes with different catalyst-modified electrodes. Error bars represent the standard deviation, obtained by repeating the experiment twice. Reaction condition: [catalyst] = 0.5 $g \cdot L^{-1}$, [PMS] = 0.8 mM, [4-CP] = 0.05 mM.

In the galvanic cells experiment, two CoNC-MSi/PMS systems achieved 100% 4-CP degradation after 6-hour, while the CoNC-Si/PMS system showed only 60% 4-CP removal due to its non-ETP- dominated decontamination process. The operation time lasted for 6 hours because of the sluggish current transfer (current of ~14 nA) and slower ions migration through the salt bridge.

Agilent 34970A data acquisition switch was adopted to record the current change of the dual-chamber galvanic cell in response to 4-CP and PMS addition. The results show a sharp rise and rapid decline of current after adding 4-CP into the CoNC-MSi1 system, indicating a strong adsorption and electronic interaction between 4-CP and the catalysts. After further adding PMS, the current increased sharply again and held relatively stable thereafter, indicating a continuous reaction between the surface-bounded PMS and 4-CP mediated by the catalyst. In contrast, the CoNC-MSi2 group showed similar but much weaker current changes upon subsequent addition of 4-CP and PMS, while the CoNC-Si groups exhibited lowest responses, consistent with their different catalytic and decontamination activities.

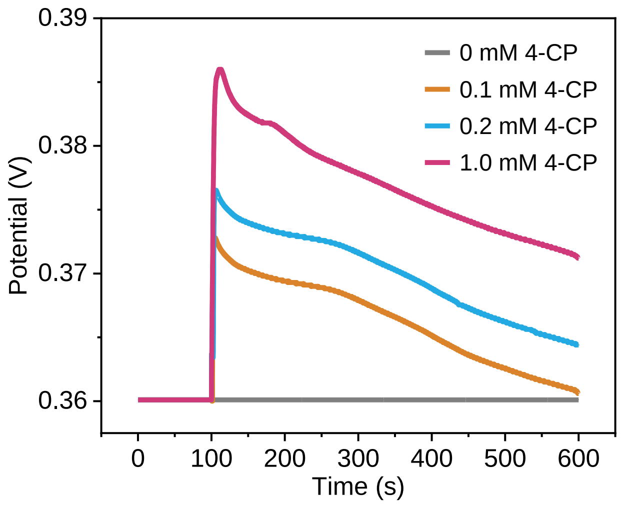

Supplementary Figure 23. Variations of open circuit potentials (OCP) of CoNC-MSi-modified electrode in the galvanic cell under different 4-CP concentrations. Reaction condition: [catalysts] = 2.5 g L^{-1}, [PMS] = 0.4 mM, [4-CP] = 0~1.0 mM, [Na_2SO_4] = 10 mM.

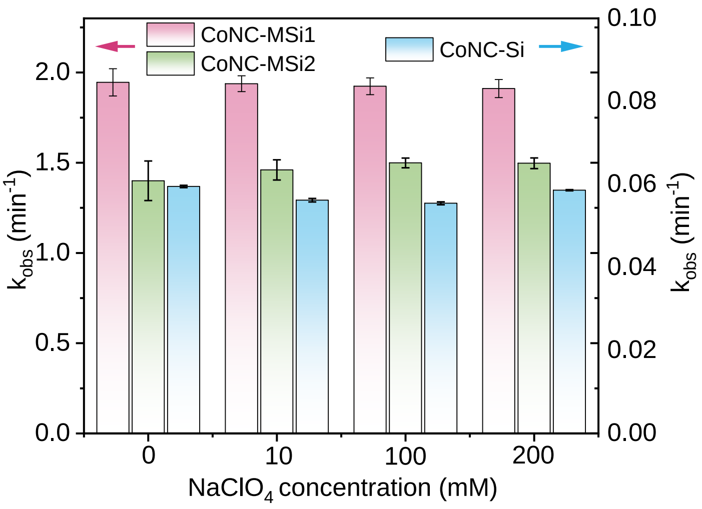

Supplementary Figure 24. Kinetic constants of 4-CP degradation in the presence of $NaClO_4$. Error bars represent the standard deviation, obtained by repeating the experiment twice. Reaction condition: [catalyst] = 0.25 $g \cdot L^{-1}$, [PMS] = 0.4 mM, [4-CP] = 0.1 mM, [$NaClO_4$] = 0~200 mM.

The increase of ionic strength could profoundly affect outer-sphere interactions (electrostatic bonding) between the solute and the material surface in both equilibrium and kinetics but not affect inner-sphere complexation (covalent bonding or a combination of covalent and ionic bonding)[32]. Therefore, the binding mode can be identified by using $NaClO_4$ to adjust the ionic strength. Here, the 4-CP degradation was almost unaffected by high-concentration $NaClO_4$, indicating an inner-sphere interaction between PMS and CoNC-MSi1.

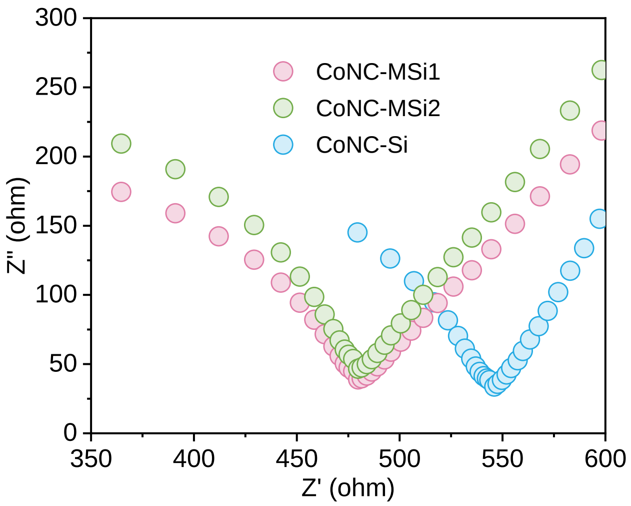

Supplementary Figure 25. EIS plots of different catalysts.

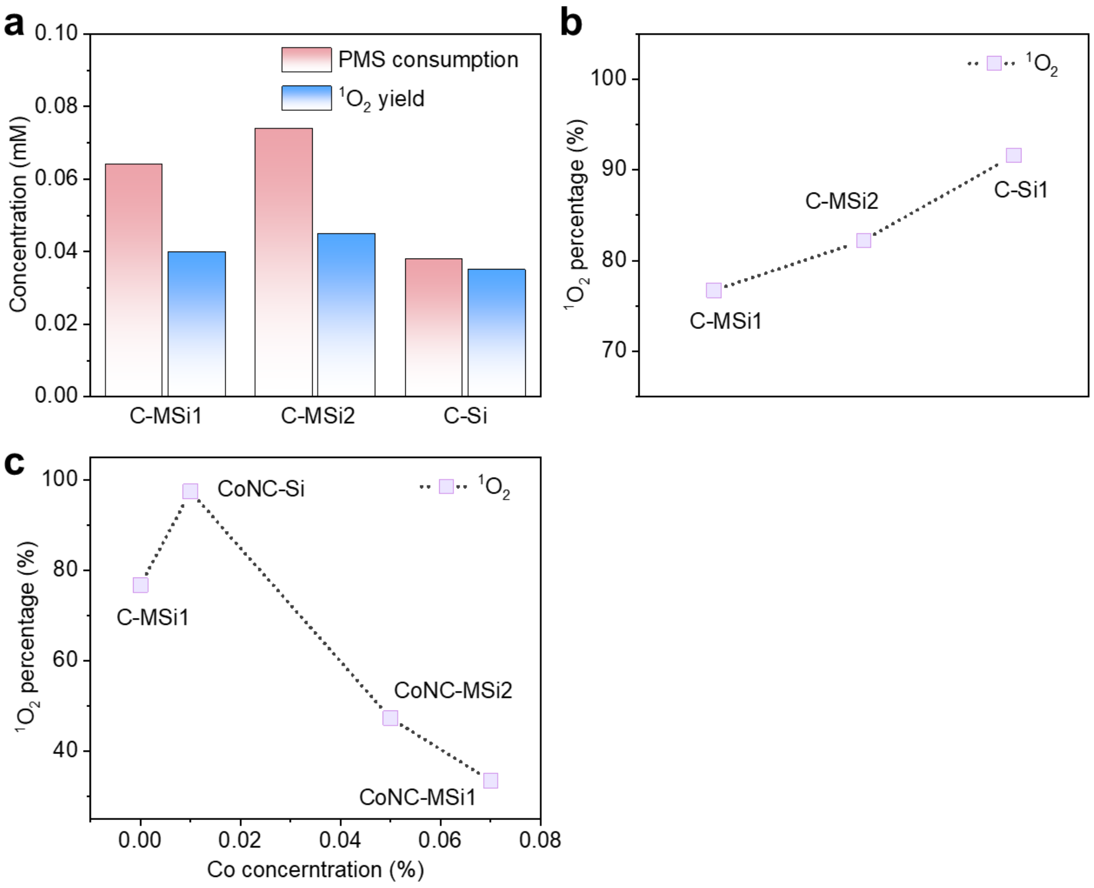

Supplementary Figure 26. Evaluating the role of $^1O_2$ species for decontamination. (a) The PMS decomposition and DPBF conversion by $^1O_2$ in three Co-free catalysts, **(b)** the $^1O_2$ percentage in different catalytic systems, **(c)** the relationship between Co concentration and persentage of $^1O_2$ generated in the catalytic system.

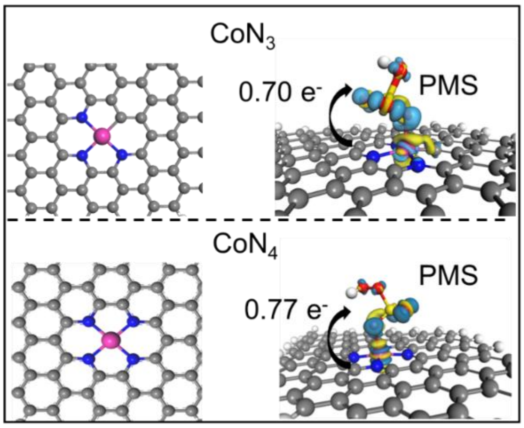

Supplementary Figure 27. The calculated charge density difference of PMS-CoNC-Si systems with different coordination numbers. The light yellow and light blue denote the electron depletion and accumulation, respectively.

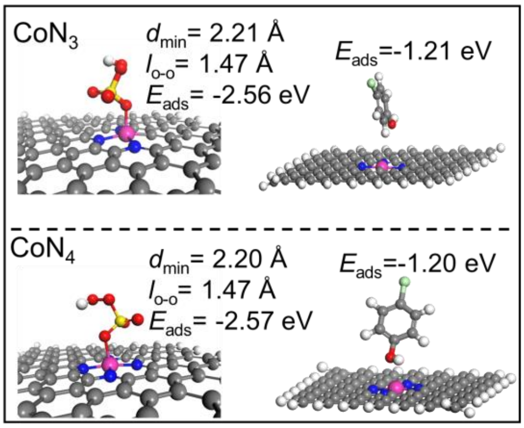

Supplementary Figure 28. Adsorption energy of PMS and 4-CP on CoNC catalyst with different N coordination numbers.

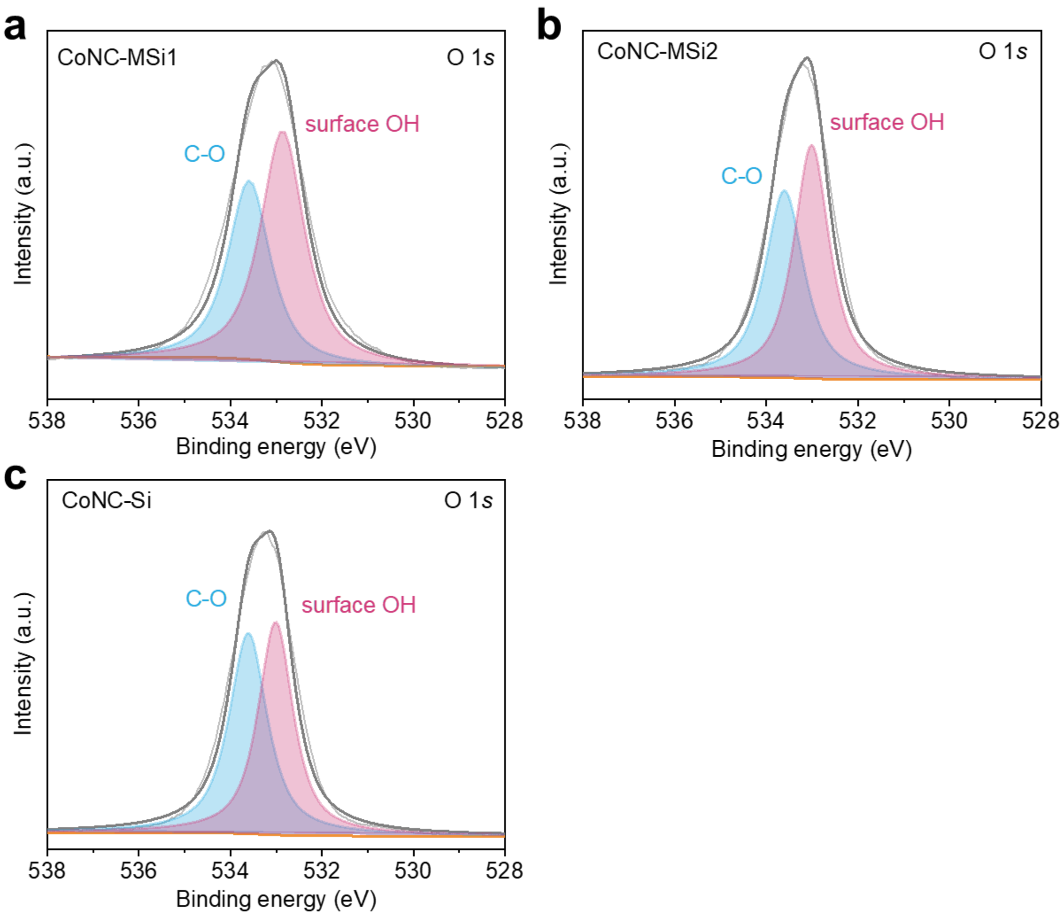

Supplementary Figure 29. O 1*s* XPS spectra of different catalysts. (a) CoNC-MSi1, **(b)** CoNC-MSi2, **(c)** CoNC-Si.

The O 1*s* XPS spectra manifest two oxygen species: the C-O (~532.8 eV) and surface -OH group (~533.6 eV)[5]. The proportion of surface -OH group increases by the order of CoNC-Si < CoNC-MSi2 < CoNC-MSi1.

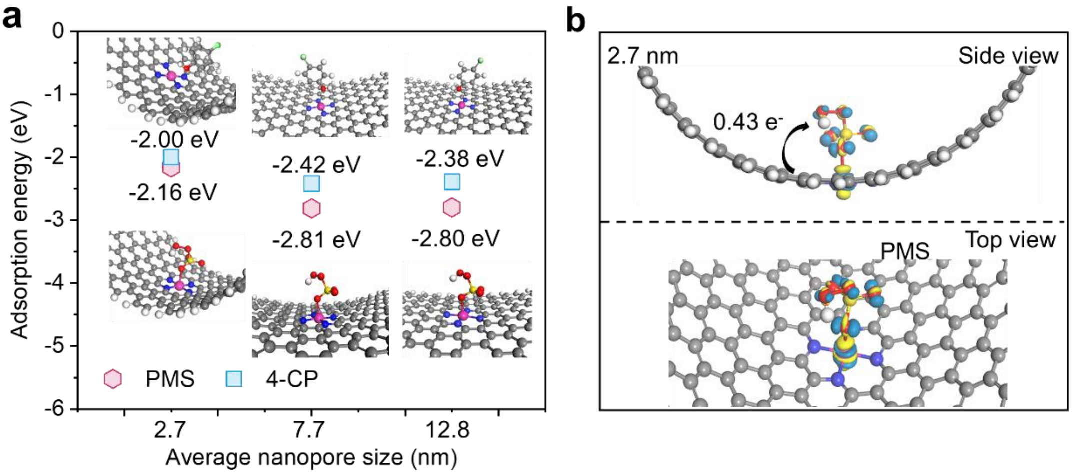

Supplementary Figure 30. Adsorption energy and electron transfer number of the nanoconfined catalysts. **(a)** Optimized adsorption structures and corresponding adsorption energy of PMS and 4-CP on nanoconfined catalysts with different pore sizes, **(b)** Charge density difference for PMS adsorption on the catalysts with 2.7 nm pore size (the light yellow and light blue denote the electron depletion and accumulation, respectively).

The medium nanopore size of CoNC-MSi1 (7.7 nm) exhibits the strongest nanoconfinement effect in Fenton-like reactions compared to those with smaller (2.7 nm) or larger (12.8 nm) pore sizes. Specifically, it has its highest adsorption energy for both PMS and 4-CP and the largest charge transfer number from catalyst surface to the adsorbed PMS (Supplementary Table 7).

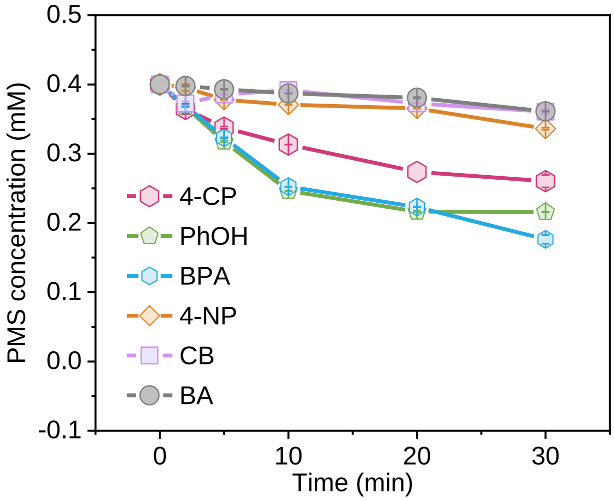

Supplementary Figure 31. PMS decomposition for degradation of different pollutants in CoNC-MSi1/PMS system. Error bars represent the standard deviation, obtained by repeating the experiment twice. Reaction condition: [catalyst] = 0.25 g·L^{-1}, [PMS] = 0.4 mM, [pollutant] = 0.1 mM.

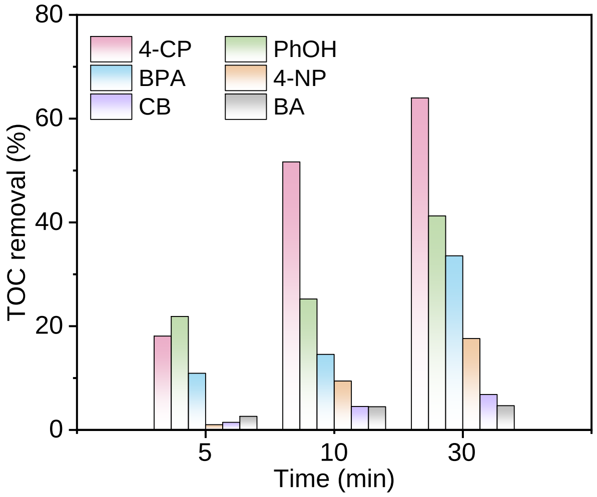

Supplementary Figure 32. Total organic carbon (TOC) measurement. TOC removal efficiency of typical electron-rich (4-CP, PhOH, and BPA) and electron-deficient pollutants (4-NP, CB, and BA) by the CoNC-MSi1/PMS system. Reaction condition: [catalyst] = 0.25 g·L^{-1}, [PMS] = 0.4 mM, [pollutant] = 0.1 mM.

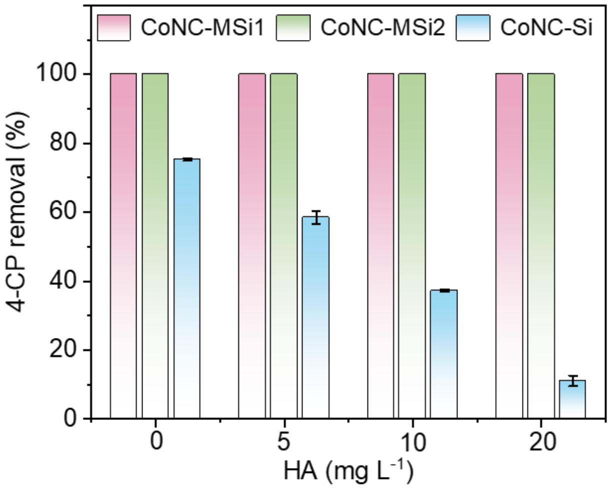

Supplementary Figure 33. Effect of humic acids (HA) on 4-CP degradation. Error bars represent the standard deviation, obtained by repeating the experiment twice. Reaction condition: [catalyst] = 0.25 g · L^{-1}, [4-CP] = 0.1 mM, [PMS] = 0.4 mM, [HA] = 0~20 mg L^{-1}.

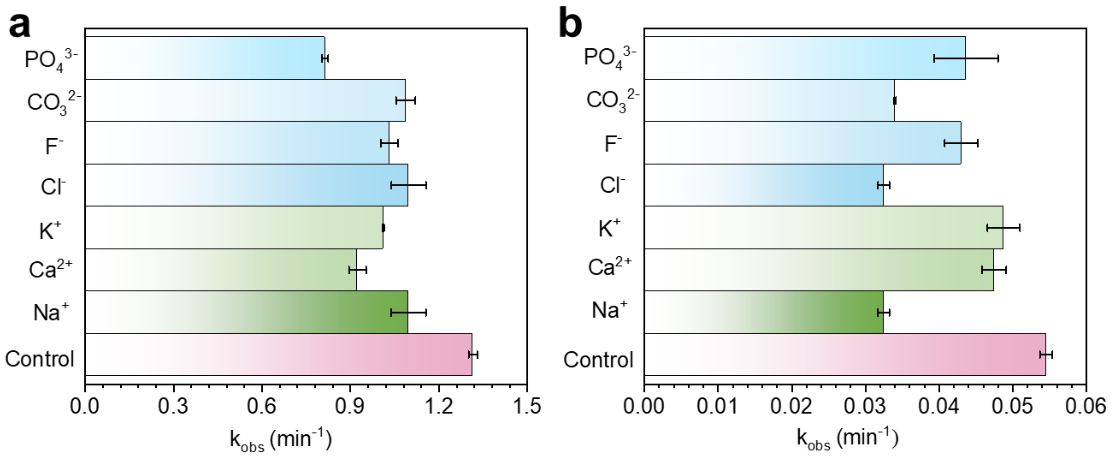

Supplementary Figure 34. Kinetic constants of 4-CP degradation in different catalytic systems in the presence of environmentally-relevant ions. (a) CoNC-MSi2 /PMS system and **(b)** CoNC-Si /PMS system. Error bars represent the standard deviation, obtained by repeating the experiment twice. Reaction condition: [catalyst] = 0.25 $g \cdot L^{-1}$, [PMS] = 0.4 mM, [4-CP] = 0.1 mM, [cations] = [anions] = 5 mM.

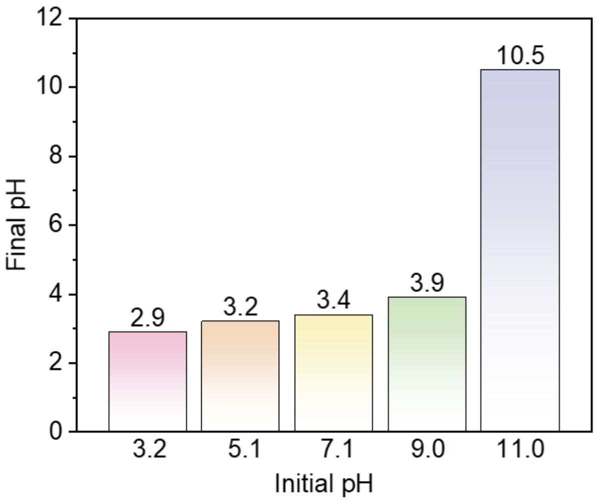

Supplementary Figure 35. Variations of pH condition in the CoNC-MSi1 catalytic system during reaction.

Reaction condition: [catalyst] = 0.25 g · L^{-1}, [PMS] = 0.4 mM, [4-CP] = 0.1 mM, initial solution pH = 3.2~11.0.

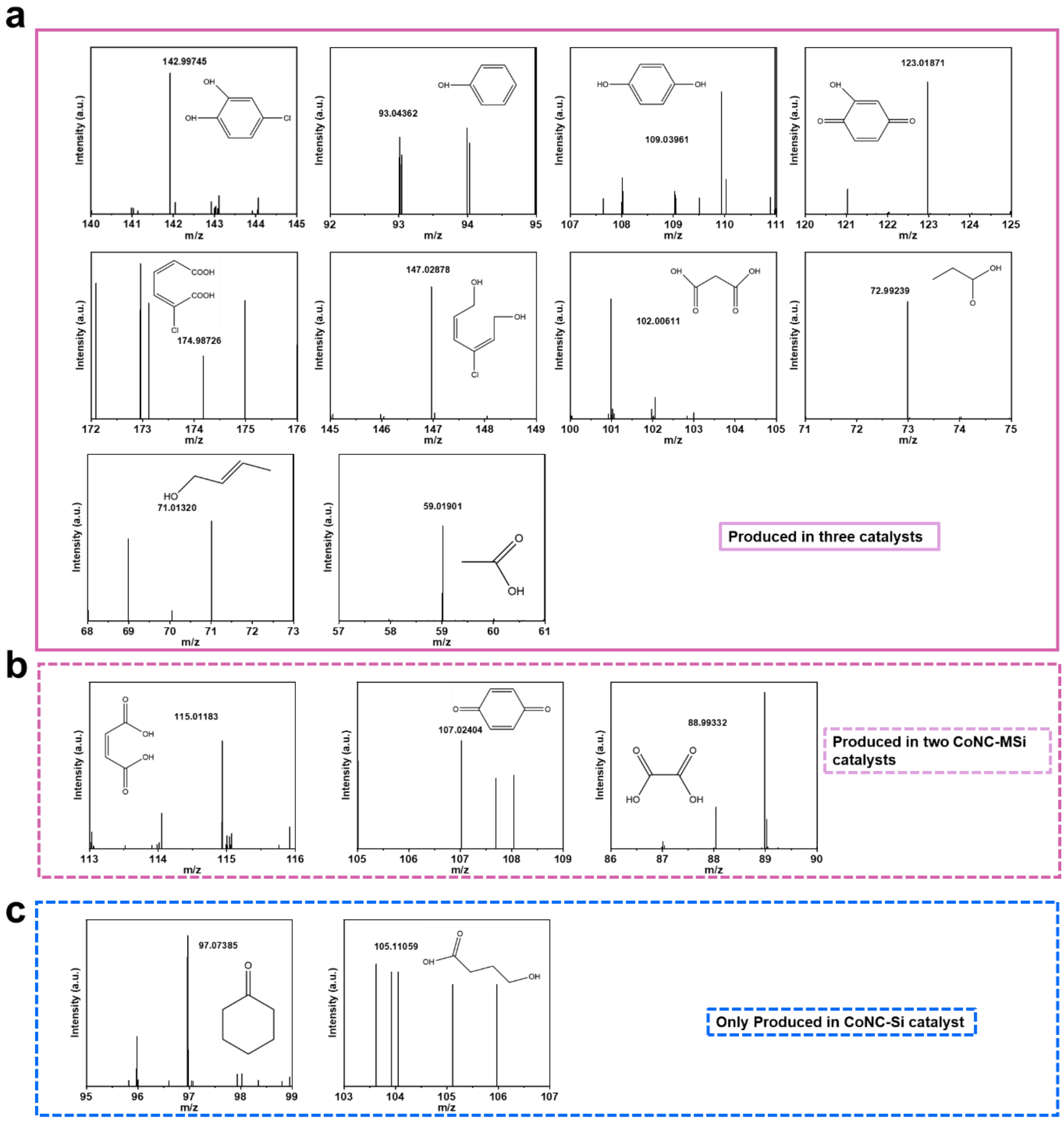

Supplementary Figure 36. 4-CP degradation intermediates detected in the catalytic systems. (a-c) HPLC-MS chromatogram (ESI negative ion mode) and corresponding molecular ion mass spectra of 4-CP degradation intermediates in the different catalytic systems. Reaction condition: [catalyst] = 0.25 g·L^{-1}, [PMS] = 0.1 mM, [4-CP] = 0.4 mM.

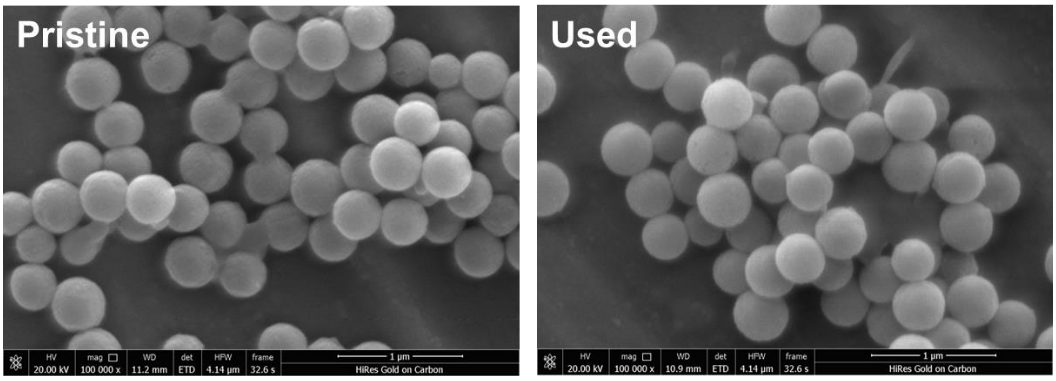

Supplementary Figure 37. SEM images of the pristine and used CoNC-MSi1 catalyst.

Note: The SEM images show unchanged morphology of the CoNC-MSi1 catalyst after reaction, indicating its high structural stability.

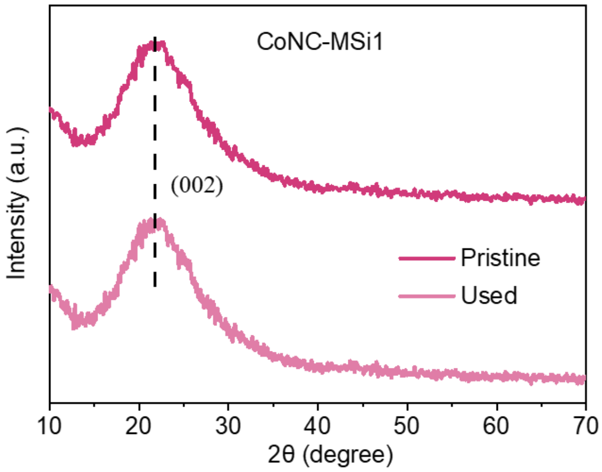

Supplementary Figure 38. XRD patterns of the pristine and used CoNC-MSi1 catalyst.

The XRD patterns show no discernable changes in the characteristic peaks of CoNC-MSi1 after reaction, indicating its good chemical and structural stability.

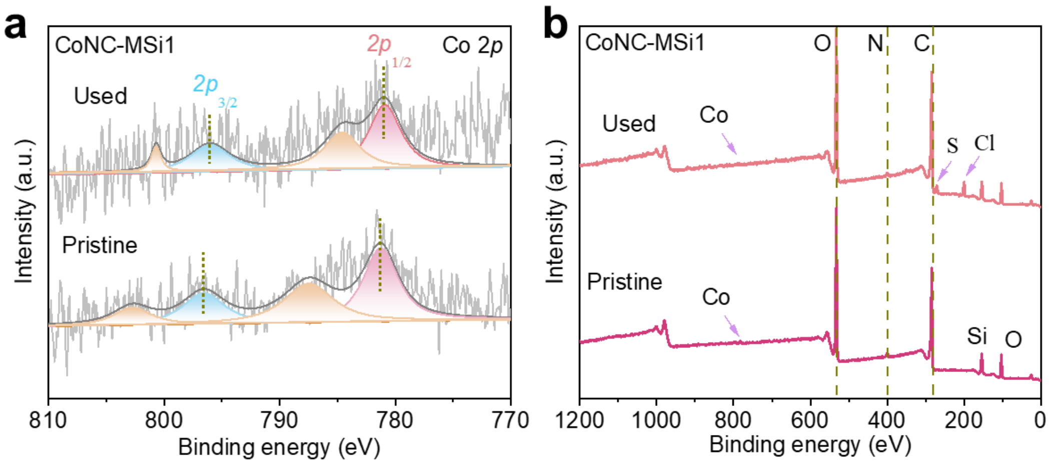

Supplementary Figure 39. XPS spectra measurement of the pristine and used catalyst. (a) Co 2*p* XPS spectra, and **(b)** full survey scans.

The Co 2*p* XPS spectra show that the peaks composition and positions were almost unchanged after reaction[33]. The peaks of Co, N, C, O, and Si in the full survey scan were also unchanged, confirming a good chemical structure stability of the CoNC-MSi1 catalyst.

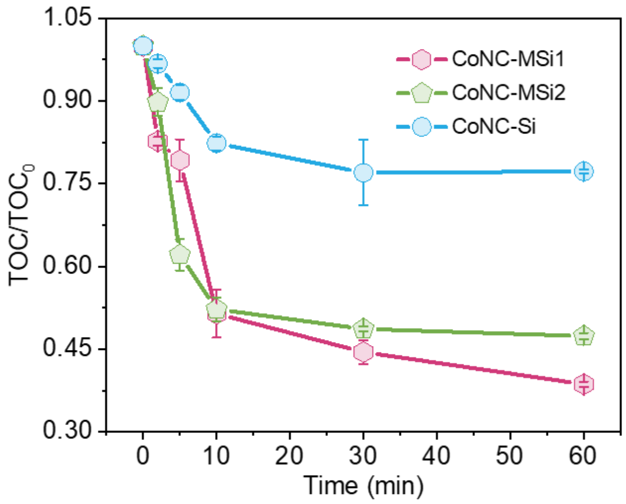

Supplementary Figure 40. TOC removal in different catalytic systems. Error bars represent the standard deviation, obtained by repeating the experiment twice. Reaction conditions: [catalysts] = 0.25 g L^{-1}, [PMS] = 0.4 mM, [4-CP] =0.1 mM.

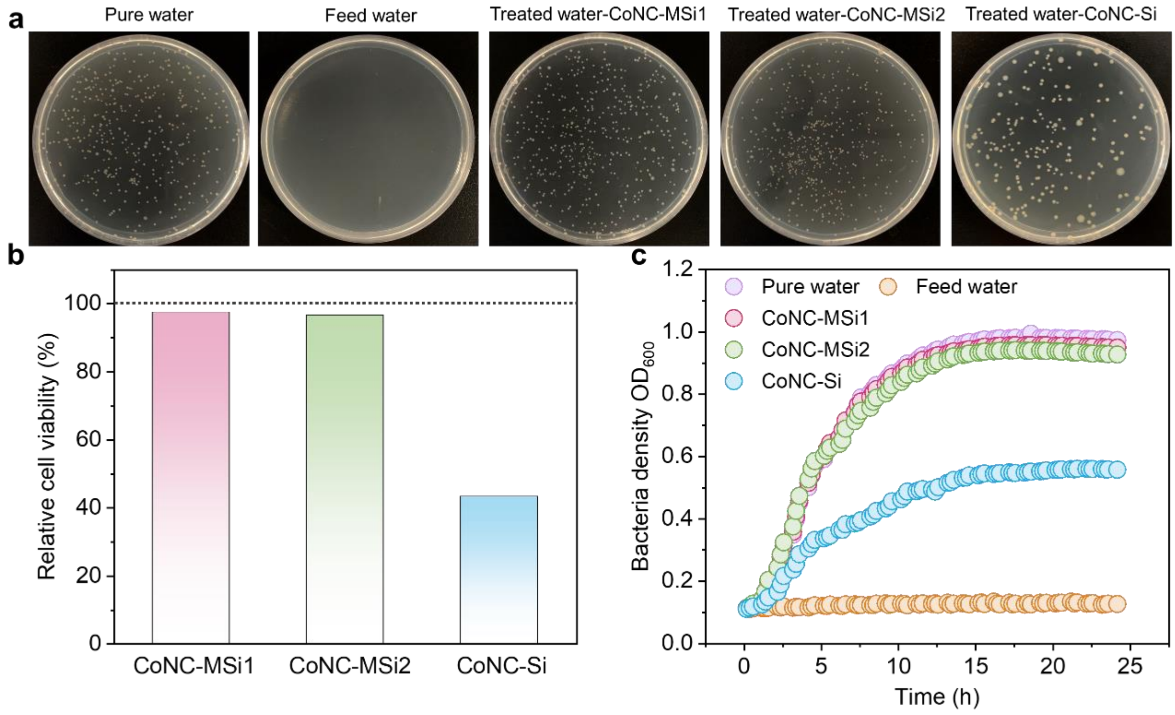

Supplementary Figure 41. Toxicity assessment of the treated water by different catalytic systems. (a) *E. coli* colony after 6-h cultivation in different water samples, **(b)** relative cell viabilities, and **(c)** growth curves.

The toxicity of the water samples was assessed by measuring the cell viability of *E. coli* bacterium after 6-h exposure[34, 35]. The results show 100% lethality of the cells under exposure to the feedwater (untreated water), confirming a high acute toxicity of 4-CP. After treatment, *E. coli* cells still remained 96% viability for the CoNC-MSi/PMS systems, indicating >90% toxicity reduction after the treatment. In contrast, only 43% toxicity reduction of the treated water was achieved by the CoNC-Si/PMS system, due to its relatively low decontamination efficiency. Therefore, the two CoNC-MSi/PMS systems show good ability of water detoxification.

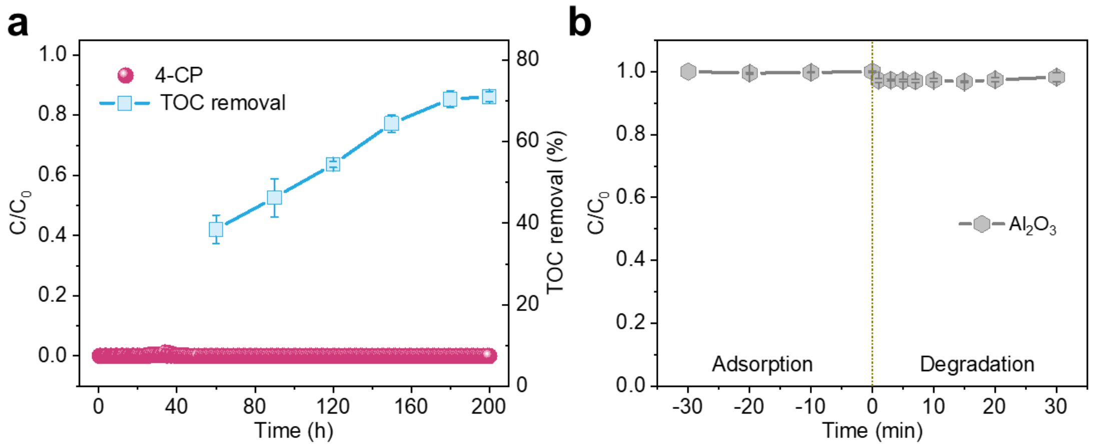

Supplementary Figure 42. 4-CP and TOC removals in different catalytic system operated in continuous-flow packed-bed reactor. (a) Co-MSi1/Al_2O_3 /PMS system, and **(b)** Al_2O_3/PMS system. Error bars represent the standard deviation, obtained by repeating the experiment three times. Reaction condition **(a)**: [catalysts] = 1.5 g, [PMS] = 0.2 mM, [4-CP] =0.05 mM, and **(b)**: [catalysts] = 0.25 g · L^{-1}, [PMS] = 0.4 mM, [4-CP] =0.1 mM (spiked in DI water).

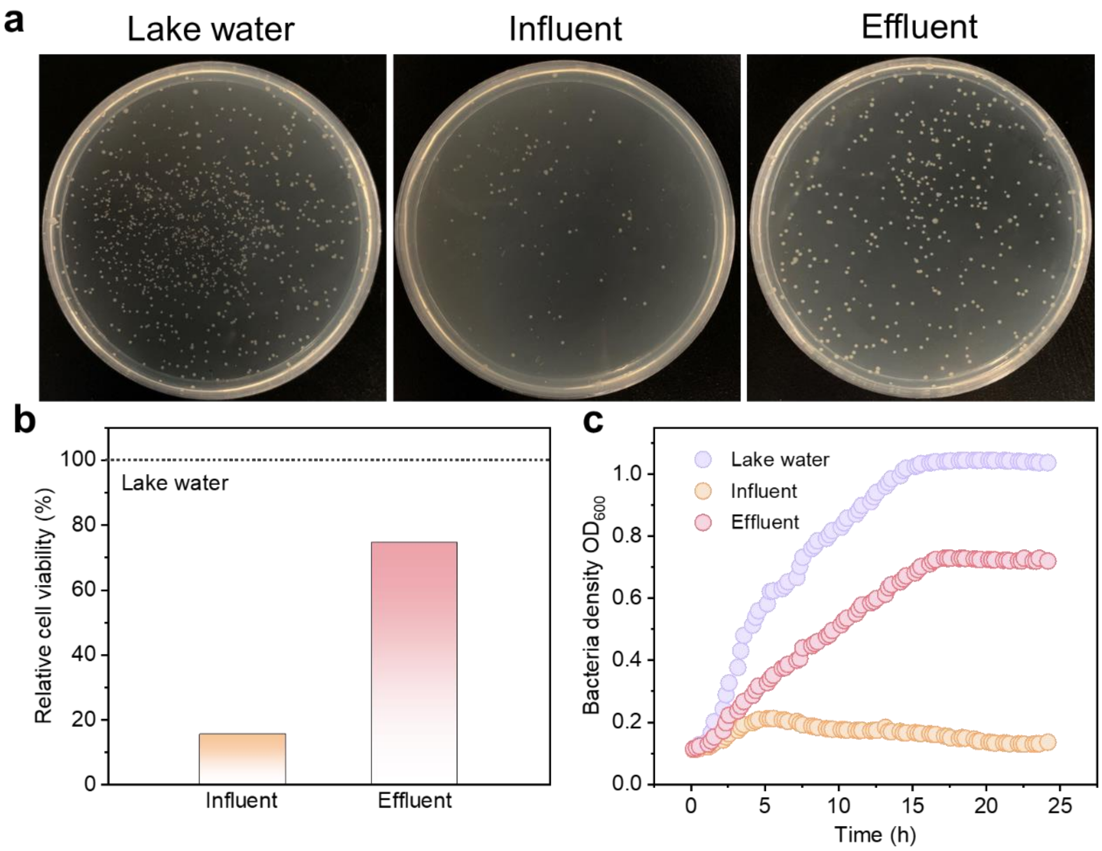

Supplementary Figure 43. Toxicity assessment in the continuous-flow reactor. **(a)** *E. coli* colony after 6-h cultivation in different water samples, **(b)** relative cell viabilities, and **(c)** growth curves in different systems.

The results confirm a drastically reduced toxicity of the water sample after treatment in the continuous-flow reactor. The toxicity reduction was consistent with the efficient 4-CP degradation, with a mineralization degree of 70% achieved, suggesting the potential of practical application in the future.

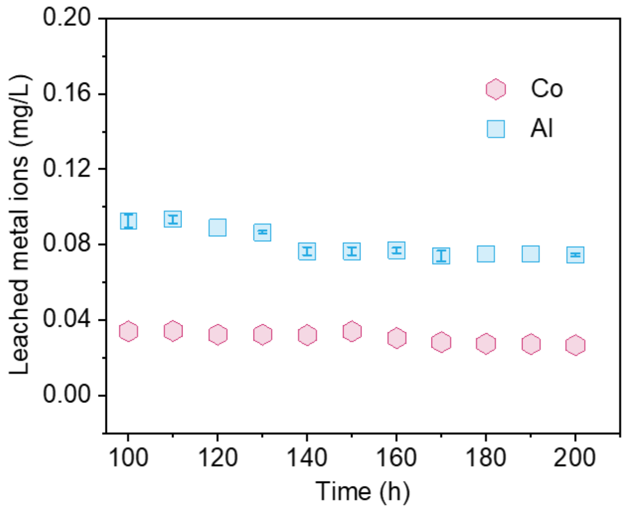

Supplementary Figure 44. Leaching amounts of Co^{2+} and Al^{3+} during continuous-flow operation for treatment of lake water. Error bars represent the standard deviation, obtained by repeating the experiment twice.

The leaching amount of Co and Al in the continuous-flow system after 200 h operation were only 0.026 and 0.075 mg L^{-1}, respectively. Both values are below the limits of "Guidelines for Drinking-Water Quality, 4th edition" (World Health Organization) and China's environmental quality standards for surface water (GB3838-2002) (Co: 1.0 mg L^{-1}, Al: 0.2 mg L^{-1}). The leached amounts of Co and Al accounted for 0.006% and 0.01% of their total contents in the fillers, indicating a strong stability and safety for water-purification applications[36].

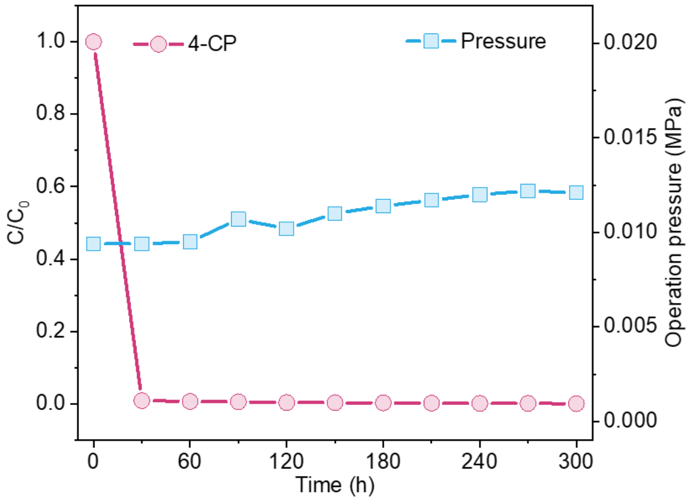

Supplementary Figure 45. The 4-CP removal and transmembrane pressure during catalytic membrane operation. Reaction condition: [PMS] = 0.1 mM, [4-CP] =0.1 mM, [flux] =200 L m^{-2} h^{-1}, and 4-CP is reached adsorption equilibrium within 2 hours before adding PMS.

When CoNC was incorporated into the inorganic ceramic membrane, the prepared catalytic membrane/PMS system can achieve ~100% 4-CP removal during 300-min reaction with an operation pressure of only 0.01±0.003 MPa, which was only ~3% of the RO membrane pressure.

Supplementary references

1. Jawad A, *et al.* Tuning of Persulfate Activation from a Free Radical to a Nonradical Pathway through the Incorporation of Non-Redox Magnesium Oxide. *Environmental Science & Technology* **54**, 2476-2488 (2020).

2. Si Y, *et al.* Reusing Sulfur-Poisoned Palladium Waste as a Highly Active, Nonradical Fenton-like Catalyst for Selective Degradation of Phenolic Pollutants. *Environmental Science & Technology* **56**, 564-574 (2022).

3. Guo Z-Y, *et al.* Electron delocalization triggers nonradical Fenton-like catalysis over spinel oxides. *Proceedings of the National Academy of Sciences* **119**, e2201607119 (2022).

4. Wei Y, *et al.* Ultrahigh Peroxymonosulfate Utilization Efficiency over CuO Nanosheets via Heterogeneous Cu(III) Formation and Preferential Electron Transfer during Degradation of Phenols. *Environmental Science & Technology* **56**, 8984-8992 (2022).

5. Chen X, *et al.* Enhanced H_2O_2 utilization efficiency in Fenton-like system for degradation of emerging contaminants: Oxygen vacancy-mediated activation of O_2. *Water Research* **230**, 119562 (2023).

6. Yin T, *et al.* Anion-exchange resin adsorption followed by electrolysis: A new disinfection approach to control halogenated disinfection byproducts in drinking water. *Water Research* **168**, (2020).

7. Wan Z, *et al.* Critical Impact of Nitrogen Vacancies in Nonradical Carbocatalysis on Nitrogen-Doped Graphitic Biochar. *Environmental Science & Technology* **55**, 7004-7014 (2021).

8. Choong Z-Y, Gasim MF, Lin K-YA, Hamidon TS, Hussin H, Oh W-D. Unravelling the formation mechanism and performance of nitrogen, sulfur codoped biochar as peroxymonosulfate activator for gatifloxacin removal. *Chemical Engineering Journal* **451**, 138958 (2023).

9. Annamalai S, Shin WS. In-situ pyrolysis of Undaria pinnatifida as a green carbo-catalyst for degradation of organic contaminants: Role of inherent N and P in the degradation pathway. *Chemical Engineering Journal* **465**, 142813 (2023).

10. Zhang D, Li Y, Wang P, Qu J, Li Y, Zhan S. Dynamic active-site induced by host-guest interactions boost the Fenton-like reaction for organic wastewater treatment. *Nat Commun* **14**, 3538 (2023).

11. Guo Z-Y, *et al.* Crystallinity engineering for overcoming the activity–stability tradeoff of spinel oxide in Fenton-like catalysis. *Proceedings of the National Academy of Sciences* **120**, e2220608120 (2023).

12. Zhao Z, Wang P, Song C, Zhang T, Zhan S, Li Y. Enhanced Interfacial Electron Transfer by Asymmetric Cu-Ov-In Sites on In_2O_3 for Efficient Peroxymonosulfate Activation. *Angewandte Chemie International Edition* **62**, e202216403 (2023).

13. Zhang L-S, *et al.* Carbon Nitride Supported High-Loading Fe Single-Atom Catalyst for Activation of Peroxymonosulfate to Generate $^1O_2$ with 100 % Selectivity. *Angewandte Chemie International Edition* **60**, 21751-21755 (2021).

14. Duan P, Pan J, Du W, Yue Q, Gao B, Xu X. Activation of peroxymonosulfate via mediated electron transfer mechanism on single-atom Fe catalyst for effective organic pollutants removal. *Applied Catalysis B: Environmental* **299**, 120714 (2021).

15. Wang Z, Wang W, Wang J, Yuan Y, Wu Q, Hu H. High-valent iron-oxo species mediated cyclic oxidation through single-atom Fe-N_6 sites with high peroxymonosulfate utilization rate. *Applied Catalysis B: Environmental* **305**, 121049 (2022).

16. Qiu X, Zhao Y, Li C, Jin R, Mutabazi E. Different peroxymonosulfate activation and utilization pathways of typical cobalt oxides, cobalt-carbon and carbonaceous composites derived from metal-organic frameworks for pollutant oxidation in wastewater. *Chemical Engineering Journal* **475**, 146234 (2023).

17. Liang X, Wang D, Zhao Z, Li T, Gao Y, Hu C. Coordination Number Dependent Catalytic Activity of Single-Atom Cobalt Catalysts for Fenton-Like Reaction. *Advanced Functional Materials* **32**, 2203001 (2022).

18. Li X, *et al.* CoN_1O_2 Single-Atom Catalyst for Efficient Peroxymonosulfate Activation and Selective Cobalt(IV)=O Generation. *Angewandte Chemie International Edition* **62**, e202303267 (2023).

19. Wang Z, *et al.* Cobalt Single Atoms Anchored on Oxygen-Doped Tubular Carbon Nitride for Efficient Peroxymonosulfate Activation: Simultaneous Coordination Structure and Morphology Modulation. *Angewandte Chemie International Edition* **61**, e202202338 (2022).

20. Li J, *et al.* Atomically dispersed Fe atoms anchored on S and N–codoped carbon for efficient electrochemical denitrification. *Proceedings of the National Academy of Sciences* **118**, e2105628118 (2021).

21. Zhao Y, *et al.* Fe_3C@nitrogen doped CNT arrays aligned on nitrogen functionalized carbon nanofibers as highly efficient catalysts for the oxygen evolution reaction. *Journal of Materials Chemistry A* **5**, 19672-19679 (2017).

22. Ellerbrock R, Stein M, Schaller J. Comparing amorphous silica, short-range-ordered silicates and silicic acid species by FTIR. *Scientific Reports* **12**, 11708 (2022).

23. Chen Z, An F, Zhang Y, Liang Z, Liu W, Xing M. Single-atom Mo–Co catalyst with low biotoxicity for sustainable degradation of high-ionization-potential organic pollutants. *Proceedings of the National Academy of Sciences* **120**, e2305933120 (2023).

24. Wang H, *et al.* Sludge-derived biochar as efficient persulfate activators: Sulfurization-induced electronic structure modulation and disparate nonradical mechanisms. *Applied Catalysis B: Environmental* **279**, 119361 (2020).

25. Fei H, *et al.* Atomic cobalt on nitrogen-doped graphene for hydrogen generation. *Nat Commun* **6**, 8668 (2015).

26. Lang Y, Wu S, Yang Q, Luo Y, Jiang X, Wu P. Analysis of the Isotopic Purity of D_2O with the Characteristic NIR-II Phosphorescence of Singlet Oxygen from a Photostable Polythiophene Photosensitizer. *Analytical Chemistry* **93**, 9737-9743 (2021).

27. Li H, Shan C, Pan B. Fe(III)-Doped g-C_3N_4 Mediated Peroxymonosulfate Activation for Selective Degradation of Phenolic Compounds via High-Valent Iron-Oxo Species. *Environmental Science & Technology* **52**, 2197-2205 (2018).

28. Su R, *et al.* Revealing the Generation of High-Valent Cobalt Species and Chlorine Dioxide in the Co_3O_4-Activated Chlorite Process: Insight into the Proton Enhancement Effect. *Environmental Science & Technology* **57**, 1882-1893 (2023).

29. Yang B, Liu H, Zhang J. High-valent metals in advanced oxidation processes: A critical review of their identification methods, formation mechanisms, and reactivity performance. *Chemical Engineering Journal* **460**, 141796 (2023).

30. Bao Y, *et al.* Generating High-valent Iron-oxo $\equiv Fe^{IV}=O$ Complexes in Neutral Microenvironments through Peroxymonosulfate Activation by Zn−Fe Layered Double Hydroxides. *Angewandte Chemie International Edition* **61**, e202209542 (2022).

31. Zhou X, Zhao Q, Wang J, Chen Z, Chen Z. Nonradical oxidation processes in PMS-based heterogeneous catalytic system: Generation, identification, oxidation characteristics, challenges response and application prospects. *Chemical Engineering Journal* **410**, 128312 (2021).

32. Zhang T, Zhu H, Croué J-P. Production of Sulfate Radical from Peroxymonosulfate Induced by a Magnetically Separable $CuFe_2O_4$ Spinel in Water: Efficiency, Stability, and Mechanism. *Environmental Science & Technology* **47**, 2784-2791 (2013).

33. Wang A, *et al.* Enhanced and synergistic catalytic activation by photoexcitation driven S−scheme heterojunction hydrogel interface electric field. *Nat Commun* **14**, 6733 (2023).

34. Yamaguchi Y, Inouye M. Regulation of growth and death in Escherichia coli by toxin–antitoxin systems. *Nature Reviews Microbiology* **9**, 779-790 (2011).

35. Rajhans G, Barik A, Sen SK, Masanta A, Sahoo NK, Raut S. Mycoremediation and toxicity assessment of textile effluent pertaining to its possible correlation with COD. *Scientific Reports* **11**, 15978 (2021).

36. Chen Y, Zhang G, Liu H, Qu J. Confining Free Radicals in Close Vicinity to Contaminants Enables Ultrafast Fenton-like Processes in the Interspacing of MoS_2 Membranes. *Angewandte Chemie International Edition* **58**, 8134-8138 (2019).
